# Parallel Immunizations of Rabbits Using the Same Antigen Yield Antibodies with Similar, but Not Identical, Epitopes

**DOI:** 10.1371/journal.pone.0045817

**Published:** 2012-12-19

**Authors:** Barbara Hjelm, Björn Forsström, John Löfblom, Johan Rockberg, Mathias Uhlén

**Affiliations:** 1 School of Biotechnology, AlbaNova University Center, Royal Institute of Technology, Stockholm, Sweden; 2 Science for Life Laboratory, Royal Institute of Technology, Stockholm, Sweden; University of Massachusetts Medical Center, United States of America

## Abstract

A problem for the generation of polyclonal antibodies is the potential difficulties for obtaining a renewable resource due to batch-to-batch variations when the same antigen is immunized into several separate animals. Here, we have investigated this issue by determining the epitopes of antibodies generated from parallel immunizations of rabbits with recombinant antigens corresponding to ten human protein targets. The epitopes were mapped by both a suspension bead array approach using overlapping synthetic 15-mer peptides and a bacterial display approach using expression of random fragments of the antigen on the surface of bacteria. Both methods determined antibody binding with the aid of fluorescent-based analysis. In addition, one polyclonal antibody was fractionated by peptide-specific affinity capture for in-depth comparison of epitopes. The results show that the same antigen immunized in several rabbits yields polyclonal antibodies with similar epitopes, but with larger differences in the relative amounts of antibodies to the different epitopes. In some cases, unique epitopes were observed for one of the immunizations. The results suggest that polyclonal antibodies generated by repeated immunizations do not display an identical epitope pattern, although many of the epitopes are similar.

## Introduction

As antibodies have proven to be an exceptional tool to study the proteins of human biology and disease the need to create well-validated reagents of this kind is evident [1]. Several initiatives have started to generate antibodies and other affinity reagents in a systematic genome-wide manner, including the Human Protein Atlas project [2], the SH2-consortium [3] and the protein binder consortiums [4]. In addition, various commercial providers have generated several hundred thousands antibodies towards human proteins, and approximately 150,000 of these antibodies are listed in the community-based Antibodypedia portal [5]. The Human Protein Atlas [6] with information on more than 11,000 protein-coding genes, contains tissue profiles for more than 60 human cell types covering 48 tissues and organs, including liver, kidney, heart, different parts of the brain, the gastrointestinal tract etc. which are based on more than 14,000 commercially available antibodies.

An important issue in this regard is the renewability of antibodies. Ideally, when results are obtained with well-validated antibodies, the reagent should be available to the scientific community indefinitely for further in-depth functional studies. This is the driving force for efforts trying to generate truly renewable affinity reagents, such as monoclonal antibodies using hybridoma cells or recombinant protein binders, such as antibody-fragments [7], scaffold binders [8] or nucleic acid based binders, such as aptamers and somamers [9]. However, more than 70% of the antibodies in Antibodypedia and 80% of the antibodies in the Human Protein Atlas are polyclonal antibodies [10]. Here, the limited availability of many polyclonal antibodies is a great concern, since there only exist a limited supply from the original immunization.

The degree of renewability of polyclonal antibodies has been questioned, due to possible batch-to-batch variations when a follow-up immunization is done to generate new quantities of antibodies. In the diagnostic arena, this problem has been over-come by immunizing large animals, such as sheep or goat, to generate large quantities of antibodies. Alternatively, many animals, such as rabbits, are immunized with the same antigen and the sera from many animals are pooled to generate a large supply of antibodies with the same batch number.

However, despite the frequent use of polyclonal antibodies, few studies have been performed in the past to estimate the degree of reproducibility when a new batch of polyclonal antibodies have been generated by immunization of a second animal. Recently, Larsson et al [11] used two recombinant antigens in repeated immunizations and determined the epitopes and immunohistochemistry staining patterns for the obtained antibodies. They concluded that all immunizations detected the correct band in Western blotting, but they rendered different staining patterns in IHC possibly related to their different epitope patterns. In the work by Geysen et al [12], the comparison of seven sera from outbred rabbits immunized with myohemerythrin, showed that no antibody specificity was common in all seven rabbits.

**Figure 1:**
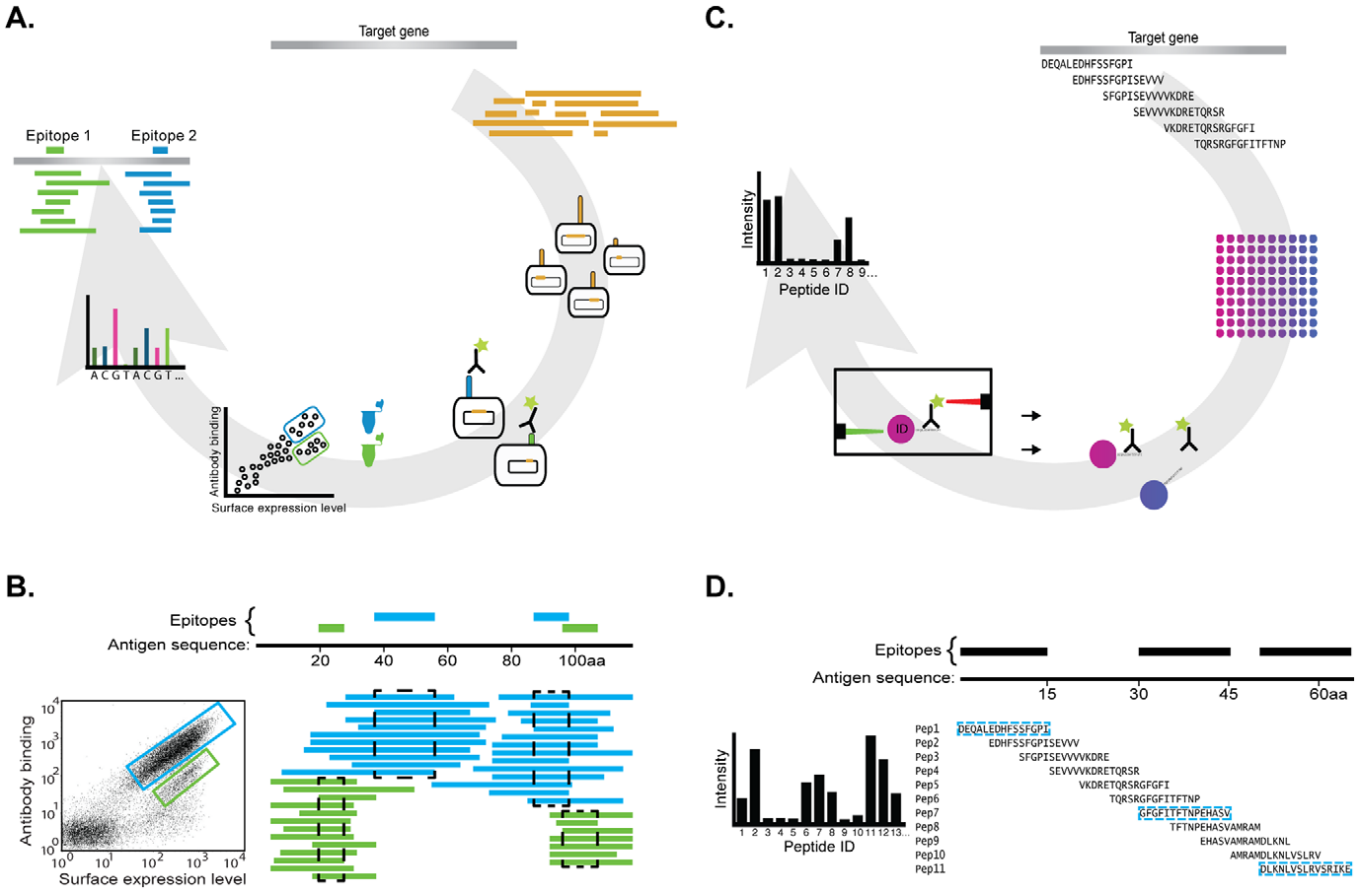
Schematic overview of the epitope mapping methods used in the study. (**A**) Epitope mapping using bacterial display, in which the target gene is fragmented and a library of clones is expressed on *S. carnosus.* The cell displayed peptide library is assayed for binding to the antibody using a flow cytometer Binding clones are sorted, sequenced and aligned back to the antigen sequence in order to conclude epitopes. (**B**) The sequencing of the flow-sorted libraries is used to determine the epitope regions. To the left, a typical FACS dot plot showing sorting of a cell displayed library incubated with the investigated antibody. The colored bars to the right show aligned binding sequences from the different sorted populations and on top the consensus epitopes derived from the alignment. (**C**) Epitope mapping using peptide bead arrays with overlapping peptides, spanning the antigen sequence, coupled to color coded beads. Antibody binding towards the peptides is evaluated using a flow cytometer instrument and epitope regions are identified on the antigen. (**D**) A schematic view of how binding profiles are used to determine epitope regions.

Established methods for epitope mapping of antibodies involves chemical synthesis of peptides [13], [14] or peptide display on phages [15], [16]. Recently, we have described two independent methods for epitope mapping of antibodies [17], as schematically outlined in Figure 1. The first method relies on bacterial surface display on *Staphylococcus carnosus* in which the gene encoding the target protein is fragmented, cloned into an expression vector and subsequently introduced into *S. carnosus* host cells (Figure 1A). A library of bacterial cells is created, each member with a small fragment of the original gene expressed on the surface of the cell. The cells are incubated with the antibody to be mapped labeled with a fluorescent dye and the cells are analyzed in a flow cytometer so that cells expressing fragments bound by the antibody can be collected. These cells are grown, the insert of the expression vector DNA sequenced and the insert is mapped back to the original gene sequence. In this way, the amino acid sequence binding to the antibody can be mapped back in an efficient manner. The second method relies on suspension bead arrays with color-coded beads, in which each has a synthetic peptide bound to its surface (Figure 1C). The bead mixture with overlapping peptides spanning the whole antigen sequence is incubated with the fluorescently labeled antibody and the beads are analyzed on a flow sorter capable of identifying each color-coded bead. Both methods will give an “apparent affinity” of the binding to the corresponding epitope, i.e. a signal corresponding to the amount of bound antibody. However, it should be noted that since the antibody is polyclonal, the signal is dictated both by the affinities as well as the amount of antibodies directed to the particular epitope in the polyclonal mixture. The latter method relies on binding to linear epitopes, while the bacterial surface display method should be able to identify longer structural epitopes.

Here, we have used these two independent methods to investigate the batch-to-batch variations of polyclonal antibodies by systematically exploring the antibody binding patterns when the same antigen is immunized into separate animals. Ten recombinant antigens corresponding to protein targets of different types and functions have been selected to explore the reproducibility of one immunization towards another in terms of epitope pattern and Western blotting. The results have implications for efforts to generate a renewable resource of affinity reagents covering the complete human proteome.

## Results

### 

#### The protein targets used in the study

We have investigated antibodies targeting ten human proteins, including proteins from diverse protein families, such as cell surface proteins, nuclear proteins and mitochondrial proteins. In all cases, a recombinant protein fragment (PrEST) was expressed in *Escherichia coli* as a fusion protein to a N-terminal hexa-histidine tag followed by a solubilizing protein fragment, the albumin binding protein (ABP) region of streptococcal protein G [18]. The size of the antigen in each immunization is listed in Table 1, which on average consist of 120 amino acids, excluding the fusion tag. The recombinant proteins were affinity purified using IMAC and the size of the fusion protein was confirmed by mass spectrometry. The antigens were used to immunize separate New Zealand White rabbits, the sera were collected and the polyclonal antibodies were purified using the respective antigen as ligand. As shown in Table 1, all antigens were immunized in three separate animals.

**Table 1:** The protein targets and the antigens used in the study.

Gene	Description	Mw for protein target (kDa)	No of aa for antigen	No of immunizations	Epitope mapping
1. HNRNPH2	Heterogeneous nuclear ribonucleoprotein H2	49,3	115	3 (2)	SP+BD
2. SYNJ2BP	Synaptojanin 2 binding protein	15,9	112	3	SP+BD
3. RPS6KA5	Ribosomal protein S6 kinase, 90 kDa, polypeptide 5	89,9	136	3	SP+BD
4. ERBB2	Erythroblastic leukemia viral oncogene homolog 2 (Her2)	137,9	96	3	SP+BD
5. TYMP	Thymidine phosphorylase	50,4	125	3	SP+BD
6. PDXP	Pyridoxal (vitamin B6) phosphatase	31,7	137	3	SP+BD
7. FBXO28	F-box protein 28	41,1	101	3	BD
8. C22orf29	Chromosome 22 open reading frame 29	39,3	125	3	BD
9. FOXP2	Forkhead box P2	79,9	104	3	BD
10. IL17RA	Interleukin 17 receptor A	96,1	128	3	BD

Gene name and description of protein targets are listed as denoted by ENSEMBL. The molecular weight (kDa) of the protein target is shown. If several splice variants, the isoform with the largest molecular weight is shown. Further, the number of amino acids of the antigen (PrEST) is shown. The number of immunizations and the method of epitope mapping used in each cases is finally listed, with SP representing synthetic peptides using the suspension bead array method and BD representing bacterial surface display using gene fragment expressed on the surface of *Staphyloccoccus carnosus* cells.

### Functional studies of the antibodies obtained from the repeated immunizations

**Figure 2:**
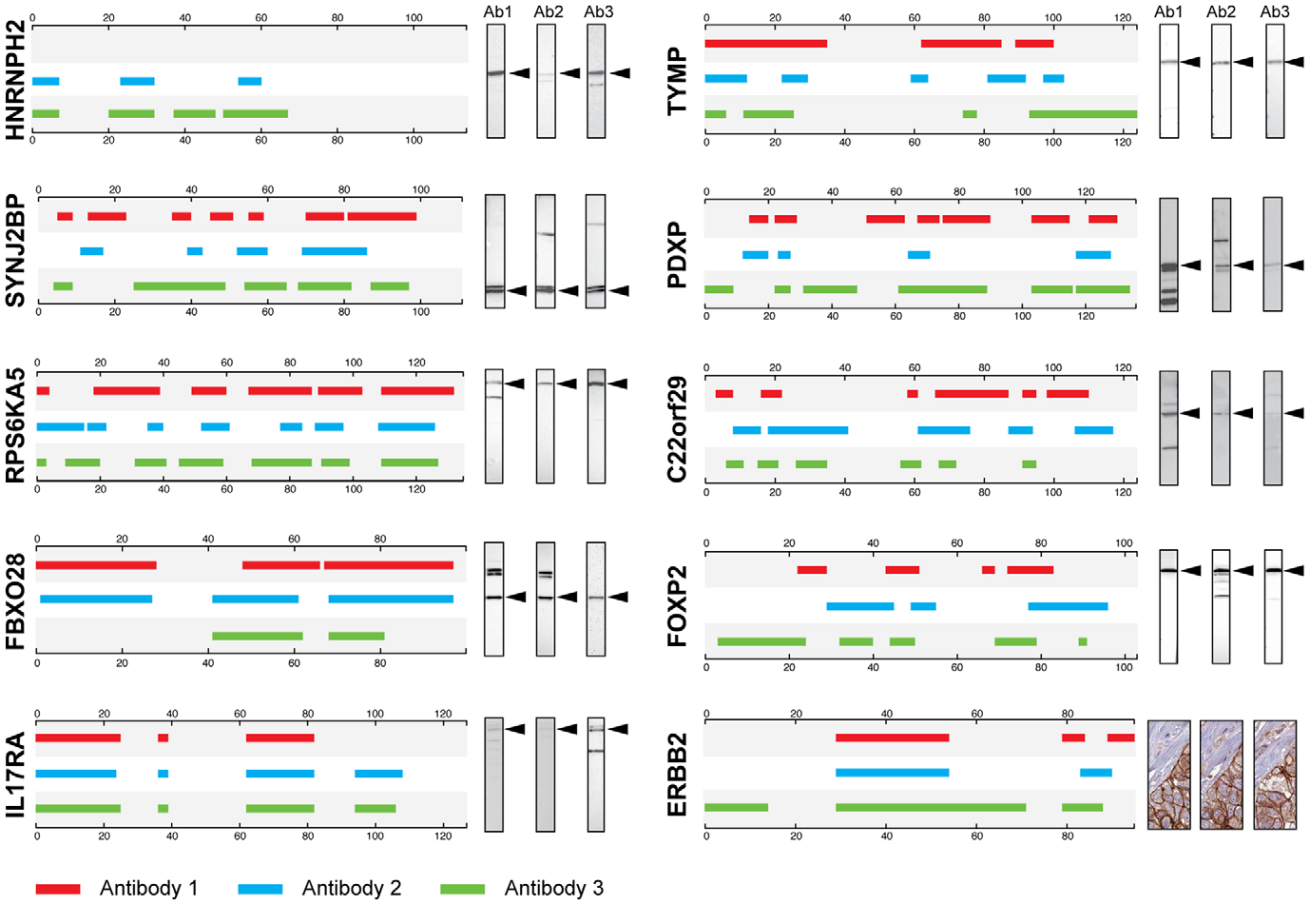
Epitope mapping of antibodies using bacterial display. Ten different bacterial displayed libraries were used to epitope map antibodies raised with the same antigen in different animals. Each box represents the different antigens with the position of epitopes corresponding to three antibodies (red, blue and green respectively). The scales on the boxes indicate amino acid position in antigen sequence. To the right of each box, the results from Western blot analysis are shown for each antibody with an arrow indicating the size of the expected target protein. An immunohistochemistry staining of breast cancer tissue is shown for the antibodies towards ERBB2, since none of the antibodies were functional in the Western blot assay.

All antibodies were validated for protein binding in Western blots of appropriate lysates or tissues (see methods for details). The results summarized in Figure 2 show that a band of correct size can be observed for all protein targets except the antibodies generated against the HER2 receptor (ERBB2). The lack of detected binding of the HER2 antibodies might be the difficulties to analyze large membrane proteins on the Western blots. Instead, immunohistochemistry of human breast cancer tissues was used and all three antibodies showed specific membrane binding to the cancer cells (Figure 2). The results show that the antibodies are specific for the intended target in a relevant functional assay. However, as shown by the Western blot analysis, some additional bands were also observed for some of the antibodies, e.g. the antibodies towards PDXP and FBXO28. This suggest that functional polyclonal antibodies are obtained by the repeated immunizations in all cases, but that some of the antibodies also show possible off-target reactivity in Western blot or potential binding to aggregates of the target antigen. Another possibility of cross-reactive background could be the use of His-tagged recombinant antigens, which may not fold as the native protein and lead to epitope exposure changes during immunization.

### Epitope mapping of antibodies using bacterial surface display

Bacterial surface display libraries were separately generated for all ten antigens listed in Table 1. The libraries were used for epitope mapping of the antibodies obtained from separate immunizations of the corresponding antigens. For all protein targets, except HNRNPH2, three antibodies from three separate immunizations were analyzed for each antigen. The results of the epitope mapping using the cell display method are shown schematically in Figure 1. To simplify, consensus epitopes have been outlined of all binding epitopes, leaving out the information of apparent affinity in between the epitopes (as seen in Figure S1, Figure S2, Figure S3, Figure S4, Figure S5, Figure S6, Figure S7, Figure S8, Figure S9, Figure S10). A list of all epitope regions is found in Supplementary Table S1. As shown in Figure 2, the epitope patterns are different in degree of similarity for the targets. For protein target IL17RA the only difference between the animals is the lack of one epitope for antibody 1. Likewise antibody 3 has one more epitope than antibody 2 for HNRNPH2 and antibody 3 of ERBB2 has an extra N-terminal epitope compared with the two other antibodies. Many of the epitopes are slightly shifted in length and direction, but locate to the same region in the antigen sequence. This is seen for example in between all three antibodies directed towards RPS6KA5, FBXO28, PDXP and ERBB2. In summary, the results show a similar, but not identical epitope pattern for all the ten protein targets analyzed.

### Epitope mapping of antibodies using suspension bead arrays

We decided to determine antigenic determinants of the antibodies against six of the targets with an independent assay (Table 1). Therefore, 15 amino acid long synthetic peptides, covering the entire antigen sequence in an overlapping manner, were immobilized to separate color-coded beads as described before [19]. A sliding window of five amino acids was used, thus each amino acid in the antigen sequence is contained in three separate peptides, except for the N- and C-terminal amino acids. All beads coupled to peptides corresponding to a particular antigen were mixed together and used for the epitope mapping. In this way, six separate bead libraries were generated each with between 17 and 26 IDs of separate color-coded beads.

**Figure 3:**
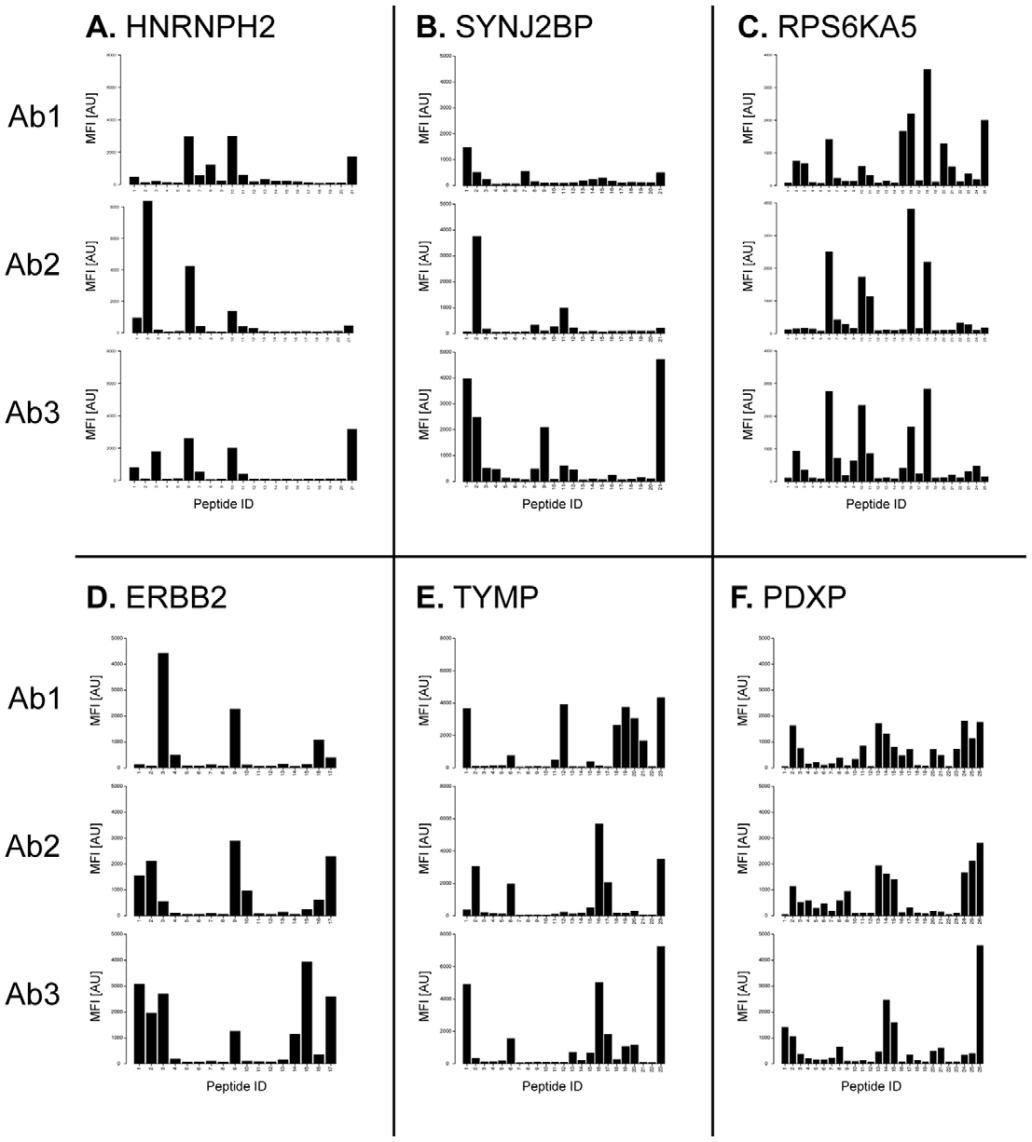
Epitope mapping of antibodies using peptide suspension bead arrays. Six different peptide arrays (A–F) were used for mapping three antibodies each (Ab1–Ab3) generated in separate animals. For each target protein, binding profiles of 15-mer peptides with a 10 amino acid overlap, are aligned on top of each other. The x-axis indicates the peptide ID and the y-axis indicates the mean fluorescence intensity (MFI).

The bead mixture for a particular protein antigen was incubated separately with the antigen-specific antibodies and analyzed on a Luminex FlexMap 3D instrument. The mean fluorescence intensity (MFI) reflecting the binding interaction was determined and plotted for each peptide. In Figure 3, the results for the six targets are shown with the signal intensities of antibody binding to each peptide shown for the three separate immunizations. A list of epitope regions is found in Supplementary Table S1.

The HNRNPH2 protein belongs to the subfamily of ubiquitously expressed heterogeneous nuclear ribonucleoproteins (hnRNPs). These proteins are annotated by RefSeq to be associated with pre-mRNAs in the nucleus and appear to influence pre-mRNA processing and other aspects of mRNA metabolism and transport. The hnRNP proteins have distinct nucleic acid binding properties and the three antibodies generated here show nuclear immunohistochemical staining in all tissues investigated (48 organs and tissues) and distinct nuclear staining using confocal microscopy based on three human cell lines (data not shown). The epitope mappings show (Figure 3A) four major epitopes shared between all three antibodies, although the relative signal intensities of the antibodies towards different epitopes differed considerably, i.e. the antibody 2 having the N-terminal epitope as the most abundant, while this epitope is minor for the other two antibodies. Similarly, the C-terminal epitope is the major epitope for antibody 3, but in contrast a minor epitope for antibody 2.

All three antibodies generated to the synaptojanin 2 binding protein (SYNJ2BP) stain the mitochondria in three human cell lines (data not shown). Immunohistochemistry analysis with the same antibodies showed moderate to strong cytoplasmic staining with a granular pattern particularly strong in the digestive system and male genital tract (data not shown). In Western blotting of RT-4 cell lysate, a double band of size 15.9 kDa was detected for all three antibodies (Figure 2). The epitope mapping (Figure 3B) revealed a relatively variable epitope pattern, although a common epitope was found in the N-terminal part of the antigen and a major epitope was found in the C-terminal part for antibody 3. The epitope in the middle of the antigen for antibody 2 and 3 is not present for antibody 1.

The epitope pattern for antibodies to the polypeptide 5 of the ribosomal protein S6 kinase (RPS6KA5) shows four common epitopes for all three immunizations (Figure 3C). Antibody 1 detected an additional lighter band in Western blot of RT-4 cell lysate except the full-length protein at 89.9 kDa recognized by all the three antibodies (Figure 2). This antibody has several additional minor epitopes, including N-terminal epitope shared with antibody 3 and an exclusive epitope at the C-terminus.

The HER2 protein, also called ERBB2, belongs to the epidermal growth factor receptor (EGFr) family of receptor tyrosine kinases. This protein binds tightly to other ligand-bound EGF receptor family members to form a heterodimer, stabilizing ligand binding and enhancing kinase-mediated activation of downstream signaling pathways, such as those involving mitogen-activated protein kinase and phosphatidylinositol-3 kinase. All three antibodies show distinct membrane staining in immunohistochemistry of a human breast cancer tissue (Figure 2). The epitope mapping shows three distinct epitopes for the first antibody (Figure 3D). The other two antibodies generated to the same antigen show similar epitope pattern, although the N-terminal epitope is shifted to the far N-terminus of the antigen. In addition, there seem to be a fourth minor epitope in the C-terminal part of the antigen for antibody 3, not present in the two other antibodies.

The TYMP gene encodes an angiogenic factor promoting angiogenesis *in vivo* and stimulating the *in vitro* growth of a variety of endothelial cells. The antibodies stain the cytoplasm and the nucleus in several tissues, including many lymphoid cells, as revealed by immunohistochemistry (data not shown). The mapping showed an almost identical binding pattern by antibodies 2 and 3. The third epitope of antibody 1 was not present in the binding of the other two antibodies however those reagents detected two other closely located epitopes (Figure 3E).

The gene for pyridoxal phosphate (PDXP) encodes the enzyme responsible for dephosphorylation of the pyridoxal 5-prime-phosphate to generate the active form of vitamin B6 that acts as a coenzyme in maintaining biochemical homeostasis. Antibody 3 showed the least cross-reactive binding pattern with one dominant band at 31.7 kDa and a weak smaller band in a Western blot of liver tissue (Figure 2). The other antibodies did also bind to the full-length protein but exhibited a more promiscuous binding pattern with several additional bands. The two last antibodies towards this enzyme show a very similar epitope pattern with three major epitopes, while the first antibody has a more diffuse pattern including the major epitopes obtained with the other two antibodies (Figure 3F).

In summary, the epitope mapping of the antibodies generated towards the six protein targets revealed similar, but not identical epitopes, when the same antigen was immunized into separate rabbits. In all cases, most of the epitopes are similar between the different immunizations, but clear differences can also be observed, i.e. for the antibodies towards TYMP, for which antibody 1 has two unique epitopes (Figure 3E).

### Fractionation of polyclonal antibodies using peptide-specific affinity capture

**Figure 4:**
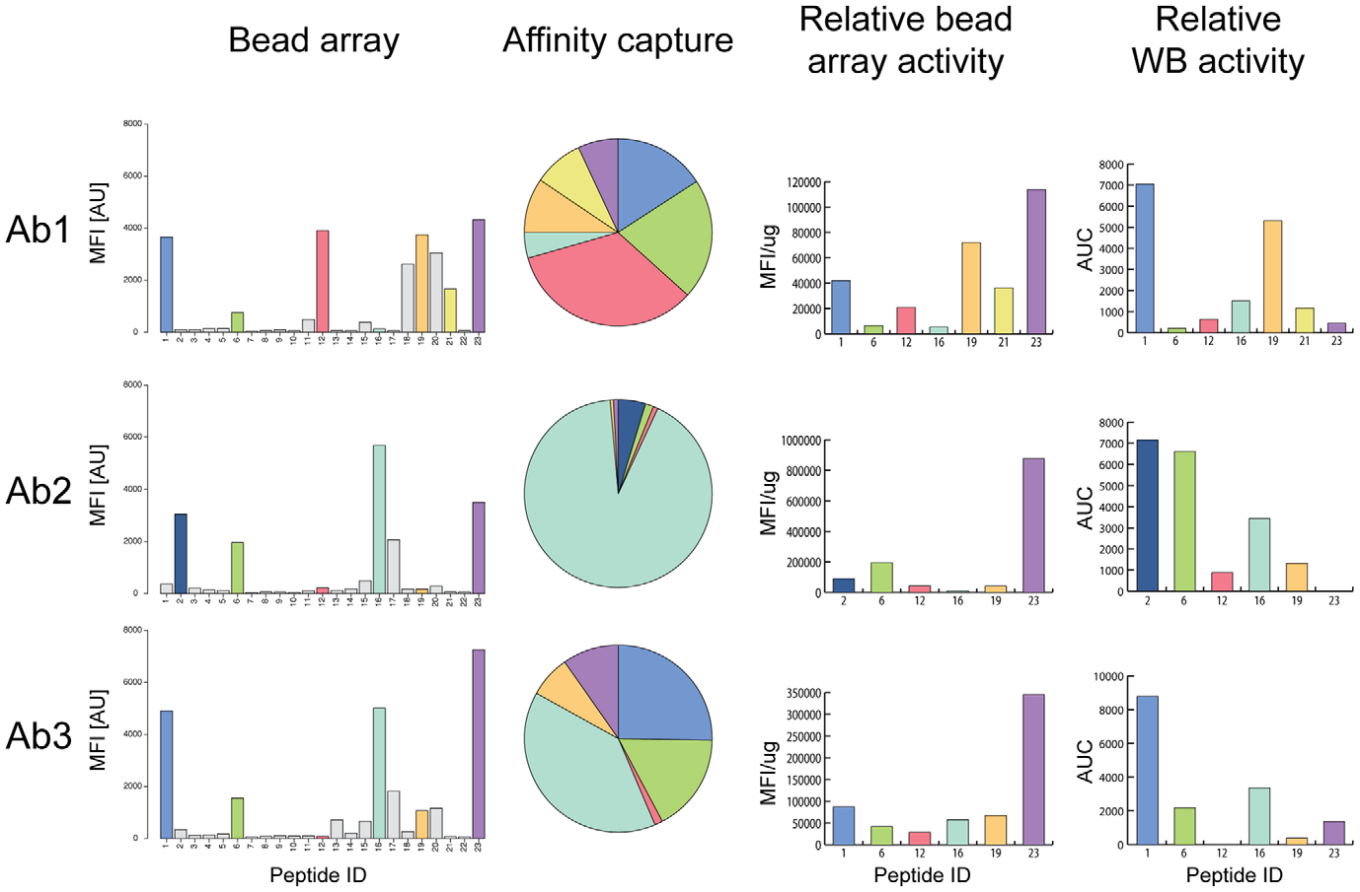
In-depth analysis of polyclonal antibodies towards TYMP. The three polyclonal antibodies from the separate immunizations of a recombinant fragment of TYMP were mapped to reveal the major epitopes, which are highlighted in different colors (Bead array). The peptides corresponding to the major epitopes were synthesized and used for affinity purification. The amount of antibodies eluted from the peptide-specific affinity capture was determined and illustrated in colors corresponding to each peptide specific antibody (Affinity capture). The “Relative bead array activity” was calculated as the mean fluorescent intensity (MFI) detected for each peptide-covered bead per µg epitope-specific antibody in the purified polyclonal antiserum. The affinity purified antibody fractions were diluted to the same concentration and subsequently used in a Western blot assay and the intensity of the correct sized band was determined using software ImageJ (Relative WB activity).

The three polyclonal antibodies from the separate immunizations of the recombinant fragment of TYMP were used for affinity capture of epitope-specific antibodies as described earlier [10]. The peptides representing the main epitopes were synthesized and used as ligands in the purification. The selected peptides are highlighted in colors in the binding profile of the antibodies to the peptide array in Figure 4 (bead array). The results show that most of the epitopes are common for the three immunizations, but a few are unique for a particular immunization, i.e. peptide 12 (pink) that is unique for immunization 1. The “Affinity capture” pie charts in Figure 4 show the relative amounts of antibodies in the different affinity purified fractions. The results reveal a dramatic difference for the relative amounts of antibodies from the different immunizations despite the fact that essentially the same epitopes are observed in the three immunizations. However, calculating “the relative bead array activity” (Figure 4) show that the functional reactivity of the antibodies in the suspension bead array for each epitope-specific antibody is relatively constant with most reactivity for antibodies towards peptide 23 and very low reactivity for antibodies towards peptide 16. The different epitope-specific fractions were subsequently assayed in a Western blot assay (Supplementary Figure S11). The results presented in Figure 4 reveals a dramatic difference in functionality for the different antibodies with highest reactivity for antibodies towards peptide 1 and low for peptide 12. The difference in relative reactivity for peptide 6 from immunization 1 and 2 suggest that the precise epitope for these two immunizations are adjacent on the same peptide, but structurally different.

#### Comparison of epitopes obtained by the two independent methods

**Figure 5:**
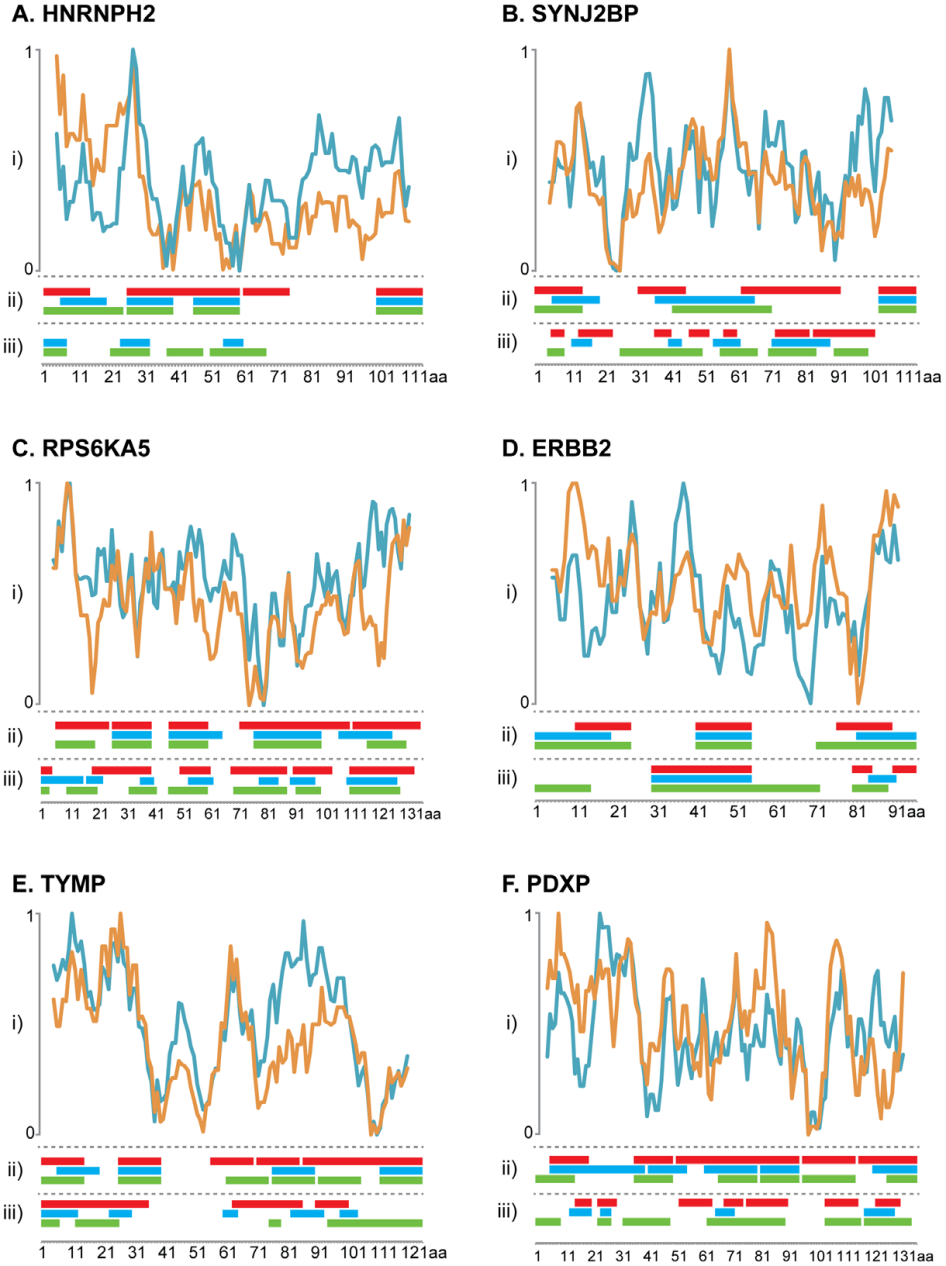
Schematic overview of the epitopes determined by the two methods and epitope prediction for the corresponding antigen. (i) Plots show the hydrophilicity value predicted by Hopp and Woods (blue) and antibody response value by Rockberg and Uhlen (orange) for the six antigens analyzed by the both epitope mapping methods. The prediction plots are illustrated above the consensus epitope sequence from the suspension bead array shown in panel (ii) and the bacterial display in panel (iii) from the different antibodies shown as colored bars (Ab1: red, Ab2: blue and Ab3: green). The x-axis indicates the amino acid position in antigen sequence and the y-axis indicates the hydrophilicity score.

The epitopes for each antibody for the six protein targets were compared between the two methods. In Figure 5, a schematic overview of the epitopes identified by the two methods is shown. Most of the epitopes are overlapping, although some epitopes are only observed using one of the two methods. Interestingly, many of the C-terminal epitopes revealed by the suspension bead approach were not detected using the bacterial display method, as exemplified by HNRNPH2 and SYNJ2BP. Due to the large window size of five amino acids for the suspension bead approach, the minimal consensus sequences are usually broader than the corresponding epitopes for the bacterial display mapping. This could be overcome by choosing a smaller window size for the suspension bead mapping.

### Analysis with epitope prediction methods

Many programs for predicting antigenicity have been published in the past, including methods such as Hoop and Woods [20] and Kyte and Dollitle [21]. Earlier studies have suggested that prediction methods based on hydrophilicity propensity scale, in which the degree of exposure of the amino acid in an aqueous solvent is calculated, has limited value [22]. Recently, Rockberg and Uhlen [23] used a comparative analysis based on 12,634 affinity-purified antibodies generated in a standardized manner against human recombinant protein fragments to compare the experimental antibody response (yield) with theoretical predictions based on 544 published propensity scales. The results show that some of the scales have some predictive power, although the overall Pearson correlation coefficient was relatively low (0.2) even for the best performing amino acid indices for the prediction of antibody amount. Based on the data set, a new propensity scale was calculated with a Pearson correlation coefficient of 0.25. In Figure 5 (panel i), the theoretical normalized propensity scales for the six antigen sequences are displayed for Hoop and Woods (blue) and the new scale from Rockberg and Uhlen (orange). As shown, the two antigen prediction methods give similar patterns, although differences can be observed in some of the regions, as exemplified by the C-terminal region of HNRNPH2. The comparisons show that there are limited overlaps between the theoretical predictions of the epitopes and the experimentally verified epitopes. However, there is a tendency that regions with low antigenicity score have low frequency of experimentally verified epitopes, as exemplified by SYNJ2BP, ERBB2 and TYMP.

### Three-dimensional structural analysis of the epitopes

**Figure 6:**
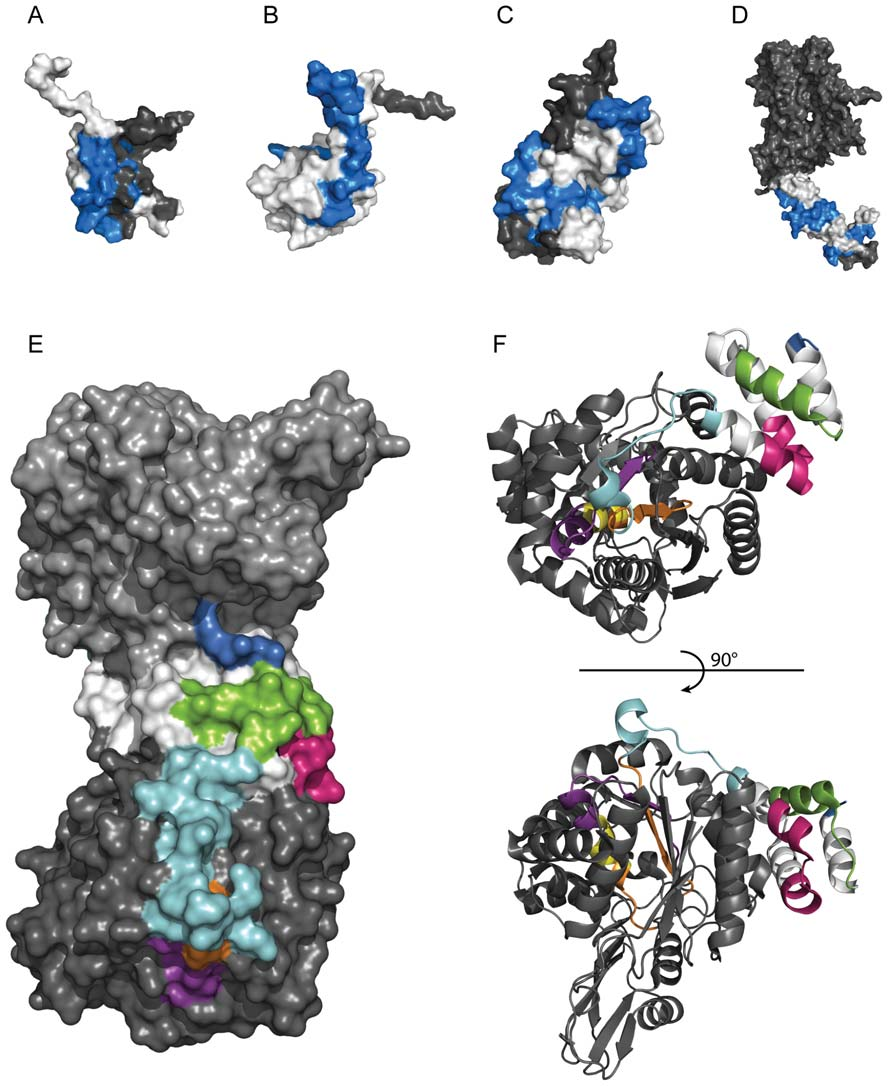
The three-dimensional structure of the epitopes. Crystal or solution structures of antigens with highlighted antigenic determinants common in all three immunizations determined by bead array mapping in blue. The molecular surface of the protein is colored grey and the antigen part is highlighted in white. The images were acquired using MacPyMOL. Structures of (**A**) RRM domain of HNRNPH2 (1wez.pdb), (**B**) PDZ domain of SYNJ2BP (2eno.pdb), (**C**) PDXP (2cfs.pdb). (**D**) extracellular domain of ERBB2 (1n8z.pdb). (**E**) Crystal structure of homodimer TYMP (2jof.pdb) showing the molecular surface with indicated epitopes (blue, green, pink, cyan, orange, yellow and purple) used for affinity purification of monospecific antibodies. (**F**), View of monomeric TYMP showing secondary structural features of epitopes.

The three-dimensional structures of some of the proteins used for design of antigens are shown in Figure 6. For the targets HNRNPH2 (A), SYNJ2BP (B), PDXP (C) and ERBB2 (D), the epitopes are shown in blue with the “epitope silent” parts of the antigen shown in white. For all four protein targets, the antibodies are directed to epitopes mainly located on the surface of the respective protein target. The epitopes of the antibodies towards TYMP (E) and (F) are also located on the surface the homodimer protein with some parts protruding into the core of the structure. Four of the epitopes are situated on the surface (blue, green, pink and cyan), one partially buried (purple) and two overlapping packed inside the protein (orange and yellow).

## Discussion

Here, we have performed a systematic study to explore the reproducibility when generating polyclonal antibodies by repeated immunizations in separate animals. Despite that a majority of the affinity reagents available to the scientific community are polyclonal antibodies, few publications are available that have investigated the difference of antibodies generated by immunizing the same antigen in a new animal (batch-to-batch variability). In addition, a lot of publications [24], [25] have stated that the lack of renewability of polyclonal antibodies calls for systematic efforts to generate monoclonal antibodies or recombinant protein binders. With this background, we have here investigated the epitope profiles of a large number of antigens immunized in several separate rabbits to generate polyclonal antibodies, taking advantage of two new epitope mapping methods to systematically analyze the binding regions of antibodies.

The bacterial display method for epitope mapping has the advantage that epitopes of different sizes can be explored and that relatively high resolution of the epitopes can be achieved by sequencing a large number of bacterial clones selected from the library. In addition, the method allows both linear and, at least in theory, conformational epitopes to be mapped. The method is relatively cumbersome, since a dedicated bacterial library is needed for each epitope mapping effort.

The suspension bead method using synthetic peptides has a more straightforward way to interpret the apparent affinities of antibodies to the different epitopes, compared to the ranking of gates in bacterial display. The method is much faster and simpler for determination of epitopes in a multiplex manner however, the synthesis of the peptides is relatively costly and the immobilization of the peptides to the color-coded beads relatively time-consuming. This has recently prompted us to develop planar micro arrays in which the peptides are synthesized using photo-litographic technologies thus enabling hundreds of thousands of peptides to be synthesized on a single micro array [26]. This has many advantages including the possibility to generate whole proteome chips covering all open reading frames of the human protein-encoded genes. In addition, higher resolution is economically feasible using overlapping peptides of only one amino acid shift and the possibility to introduce alanines and other amino acids into the peptides for exact mapping of the binding residues. Here we have used an overlap of five amino acids for our peptide mapping and obviously this means that the resolution for the binding is somewhat low (as seen in figure 5), but adequate for investigating the difference between the antibodies generated by the different immunizations.

The study has given results of both specific and general interest. Firstly, the results confirm the results obtained earlier [17] that approximately 3–5 linear epitopes are generated per 100 amino acid recombinant protein fragment, thus leaving large parts of the antigen “epitope silent”. However, as both employed methods are based on peptide mapping there is a possibility that a proportion of the antibodies are directed towards conformational epitopes and thereby not detected in this study. Secondly, the analysis of the proteins with known three-dimensional structures suggest that most of the epitopes are accessible on the surface of the native protein targets, despite the fact the recombinant protein fragments have been used for the immunizations. This might imply that either the recombinant fragment has a degree of native fold in the immunized animal or that the surface residues are inherently more antigenic. The latter is somewhat supported by the propensity scale analysis (Figure 5) showing that many of the epitope silent regions are also low in predicted antigenicity both by the original Hopp and Woods [20] method, but also by the more recent propensity scale [23].

An interesting observation is the difference of antibody reactivity for different epitopes in different applications as shown for the three antibodies towards human TYMP. By combining the epitope mapping with epitope-specific affinity capture to generate epitope-specific antibodies from the polyclonal antisera, it was possible to determine a “relative reactivity” of each fraction. This was done by dividing the immune-reactive signal intensity by the amount of antibodies in each fraction (Figure 4). The results suggest that antibodies towards the C-terminal peptide 23 (purple) have the highest apparent binding activity to the peptide epitope on beads, while the most abundant antibodies in the second and third immunizations, those towards peptide 16 (cyan), have a low specific activity. It is reassuring that all three antibodies show similar relative bead array activity for each epitope despite the fact that very different relative amounts are observed for the epitope-specific antibodies generated by the separate immunizations. The results from the Western blots show a remarkable difference in reactivity with a very low specific activity for the peptide 23 (purple) antibodies and instead the N-terminal peptide 1 or 2 (blue) is superior. This demonstrates that different epitopes are suitable for different applications most likely due to variations of the exposure of the epitopes.

The study shows that many of the main epitopes, in particular as investigated by the suspension bead array (Figure 3), are shared between the antibodies generated in different animals when the same antigen has been used for immunization. However, for all ten antigens, there is at least one minor epitope generated in one of the animals, which is not detected in the other animals, demonstrating that the polyclonal responses in the animals are not identical. Finally, apparent affinities of the epitopes, as investigated by the suspension bead array, show dramatic differences between the immunizations, as exemplified by antibody 1 and 3 using the protein SYNJ2BP (Figure 2B). Altogether, this shows that the same antigen immunized in several rabbits yields polyclonal antibodies with similar epitopes, but with larger differences in the apparent affinities of antibodies to the different epitopes. It would be interesting to see if this also holds in a broader comparison with immunization in other common hosts such as rat, mouse or goat. Earlier studies with peptide immunizations in mice have shown similar results where the epitope region of the antibodies shifts in length and direction [27].

The results presented here have potential consequences for efforts to generate renewable affinity reagents to proteins from various sources. We have recently described [10] that if epitope-specific antibodies are generated by affinity purification of polyclonal antibodies using synthetic peptides corresponding to the epitopes of the polyclonal antiserum, it is possible to investigate the functionality in different applications, such as Western blot, immunohistochemistry and immunofluorescent-based confocal microscopy for each epitope-specific fraction for the polyclonal antibody. The results demonstrated that many of the epitope-specific antibodies were not functional in these assay and that only a few were functional across all applications. Thus, it is not unlikely that some of the polyclonal antibodies generated with repeated immunizations in the study presented here would not be functional in individual applications.

In summary, we have performed a systematic study of epitope patterns obtained when an antigen is separately immunized into several animals. The results implies that caution should be taken when a new batch of antibodies is employed, since at least some of the minor epitopes obtained differs between the polyclonal antibodies. However, it is also clear from our study that many of the major epitopes re-appear showing that the immune system of the animals are capable of directing the response to the same epitopes. Our conclusion from these results is that polyclonal antibodies from repeated immunizations can be used for functional studies, but that thorough validation must be performed to ensure that the functionality of the new batch for a particular immune assay is the same as the original polyclonal antibody.

## Materials and Methods

### Ethics Statement

Biological material was derived from patients who have undergone surgical procedures for diagnostic and therapeutic reasons. As part of the diagnostic procedure, representative tissue material was collected and stored in the fresh frozen tissue biobank. Patients were informed by their doctors that this will be done and were asked for permission to have their tissue saved and stored in the biobank. This has been recorded in the referral form that follows with each tissue sample sent to pathology for diagnostics. Following an application to the local ethics committee at Uppsala University Hospital, we received an advisory statement that there are no objectives on an ethical ground, to utilize material from the biobank for protein profiling and antibody validation, provided that the material is anonymized.

### Generation of polyclonal sera

Antigens were designed based on sequence similarity to all other human proteins using the software PRESTIGE [7]. The designed gene fragments were amplified from a pool of RNA isolated from human tissues (Clontech Laboratories, Mountain View, CA, USA and Agilent Technologies, Santa Clara, CA, USA) and cloned into the vector pAff8c, in fusion with a hexa-histidine tag and an albumin binding protein, and expressed in Escherichia coli. The purified and validated recombinant protein fusions were used for immunization of New Zealand White rabbits. Rabbits were immunized intramuscularly with a primary immunization supplemented with Freund's complete adjuvant followed by booster immunizations at four, eight and twelve weeks together with Freund's incomplete adjuvant. The immunizations were performed by Agrisera AB (Vännäs, Sweden), Harlan Laboratories (Indianapolis, IN, USA) and Beijing Proteome Research Center (Bejing, China) [8] who granted us permission to use the resulting sera in this study.

### Epitope mapping using bacterial display of polyclonal sera

Ten different peptide libraries were generated as follows. Gene fragments of each of the target proteins were amplified by PCR separately. Each product pool (4.8 ml) was sonicated for 75 min and further polished using T4 DNA polymerase and phosphorylated using T4 DNA kinase (New England Biolabs, Ipswich, CA). The blunt-ended fragments were ligated into the vector pSCEM2 [17]. The library was then electroporated into *S. carnosus* TM300 as described previously [28]. 3.5 ng of each antibody was separately incubated with a cell aliquot of about tenfold of coverage in a total volume of 70 µl phosphate-buffered saline (PBS) with 0.1% Pluronic F108 NF surfactant (PBSP) prior wash and secondary labeling with goat anti-rabbit antibody. After washing cells were sorted on FACS. This procedure was repeated once again after amplification of binding cells. After a second sorting using FACS, cells were sequenced using Big Dye terminators (GE Healthcare Bio-Sciences AB, Uppsala, Sweden) and analysis on an ABI Prism® 3700 DNA sequencer (Applied Biosystems, Foster City, CA). The sequences were then aligned back to their respective antigen sequence.

### Epitope mapping using peptide bead arrays of polyclonal sera

Six different peptide sets were ordered (provided by Sigma-Aldrich, St Louis, MO). They were designed as N-terminally biotinylated 15-mers with a sliding window of five amino acids along the respective antigen sequence resulting in 17–26 peptides for the different targets. 2*10^5^ neutravidin coupled beads were added to 5 µg peptides in a total reaction volume of 125 µl PBS-B (PBS supplemented with 1% Bovine Serum Albumin) to associate for 60 min at room temperature in dark before washing, pooling of each beadmix and storage in storage buffer (BRE, Blocking Reagent for ELISA, Roche supplemented with 0.1% Pro-clean 400). Approximately 1000 beads per ID were incubated with antibody at a final concentration of 2 µg/ml in a volume of 100 µl of PBS-B for 60 minutes in dark under agitation. After washing, a secondary incubation with anti-rabbit IgG-PE (Jackson ImmunoResearch Laboratories, USA) at a concentration of 1 ng/µl in 100 µl PBS-B was performed in dark under agitation. A final washing was ended by adding 100 µl PBS-B and analyzed on a Luminex FlexMap 3D system.

### Affinity purification of epitope specific fractions

600 nmole biotinylated synthetic peptides corresponding to epitopes identified in the Luminex epitope mapping were each coupled to a 1 ml HiTrap™ Streptavidin HP column (GE Healthcare) according to the manufacturer's instructions. The columns corresponding to the epitopes of each serum were connected in series on a ÄKTAxpress™ (GE Healthcare) liquid chromatography system. 5–10 ml of each serum was filtered and diluted with wash buffer (PBS supplemented with 0.05% Tween-20) to a final volume of approximately 14 ml before loaded on the serially connected columns. After a brief washing step to complete the sample loading, the columns were taken apart and washed in parallel with 5 column volumes of wash buffer. A low pH elution buffer (200 mM Glycine, 1 mM EDTA, pH 2.5) was subsequently used to recover the individual epitope specific antibodies.

### Western blot

The antibodies were analyzed on western blot membranes prepared from 15 µg of total protein lysates from two different cell lines (RT-4 (DSMZ, GmbH Germany) or U-251 MG provided by Prof. Bengt Westermark, Uppsala University (Uppsala, Sweden)), liver tissue and one cell lysate of HEK293T cells over-expressing the target protein FOXP2 (LY407756, Origene Technologies). Antibodies towards HNRNPH2 and FBXO28 were validated using U-251 MG cell lysate, antibodies towards SYNJ2BP, RPS6KA5, IL17RA and C22orf29 were validated using RT-4 cell lysate, antibodies towards PDXP and TYMP were validated using liver tissue lysate and antibodies towards FOXP2 were validated using cell lysate of HEK293T cells over-expressing the target protein FOXP2. The proteins were separated according to size on precast 10–20% CriterionTM SDS-PAGE gradient gels (Bio-Rad Laboratories, Hercules, CA) under reducing conditions. The proteins were subsequently transferred to PVDF membranes using Criterion GelTM Blotting Sandwiches (Bio-Rad Laboratories, Hercules, CA) according to the manufacturer's instructions and the membranes were blocked (5% dry milk, 0.5% Tween-20, 1× TBS; 0.1 M Tris-HCl, 0.5 M NaCl) for 1 h at RT. The polyclonal antibodies were diluted 1/250 or 1/500 in blocking buffer and the epitope specific polyclonal antibodies were normalized to match the antibody concentration of their respective polyclonals. After 1 h incubation the membranes were washed 4×5 minutes in 1×TBS supplemented with 0.05% Tween20. Secondary HRP-conjugated swine anti-rabbit antibody (DakoCytomation, Glostrup, Denmark) was diluted 1/3000 in blocking buffer and the membranes were incubated 1 h. Unbound antibodies were removed with another round of washing before addition of HRP-substrate (SuperSignalVR West Dura Extended Duration Substrate, Pierce) and chemiluminescence detection was carried out using a Chemidoc CCD-camera system (Bio-Rad Laboratories). Band intensities of epitope-specific fractions on tissue lysate were analyzed using ImageJ (http://imagej.nih.gov/ij/, US National Institutes of Health, Bethesda, MD).

### Immunohistochemistry

Automated immunohistochemistry was performed on TMAs with breast cancer tissue from paraffin embedded specimens. Anonymized tissue material was retrieved from the archives of the Department of Pathology and Cytology, Uppsala University Hospital (Uppsala, Sweden). Glass slides were deparaffinized, hydrated and blocked before antigen retrieval by boiling for 4 min in Target Retrieval Solution, TRS, pH 6.0 (Dako, Glostrup, Denmark) in a decloaking chamber (Biocare Medical, Walnut Creek, CA). Slides were immunostained in an automated staining instrument, Autostainer (Dako). Primary antibodies and goat anti-rabbit/mouse peroxidase conjugated Envision (Dako) were incubated for 30 min each in room temperature. Diaminbenzidine (Dako) was used as chromogen for the development and Harris hematoxylin for counter-staining before scanning.

### Structural analysis of epitopes

The software MacPyMol was used to highlight epitope sequences in structures 1N8Z.psd (ERBB2), 2ENO.pdb (SYNJ2BP) 1D4B.pdb (CIDEB), 2CFS.pdb (PDXP) and 2JOF.pdb (TYMP).

### Data analysis

Propensity scores for the selected antigens were calculated as previously described [23]. Briefly propensity tables for Hopp and Woods [20] as well as Rockberg and Uhlén [23] were used to calculate average propensity values on a sliding window of 9 amino acids throughout the antigen sequence. Scores were normalized between zero and one and plotted along corresponding amino acid coordinates.

## Supporting Information

Figure S1 **Epitope mapping of two antibodies towards HNRNPH2 using bacterial display.** FACS dot plots to the left show second sorting of a staphylococcal displayed HNRNPH2 peptide library. Colored bars to the right show sequences of collected clones from each gating aligned to the original antigen sequence indicated above as a scale. On top of scale, consensus epitopes summarize the minimal sequence needed for binding from each gated population.
(TIF)

Figure S2 **Epitope mapping of three antibodies towards SYNJ2BP using bacterial display.** FACS dot plots to the left show second sorting of a staphylococcal displayed SYNJ2BP peptide library. Colored bars to the right show sequences of collected clones from each gating aligned to the original antigen sequence indicated above as a scale. On top of scale, consensus epitopes summarize the minimal sequence needed for binding from each gated population.
(TIF)

Figure S3 **Epitope mapping of three antibodies towards RPS6KA5 using bacterial display.** FACS dot plots to the left show second sorting of a staphylococcal displayed RPS6KA5 peptide library. Colored bars to the right show sequences of collected clones from each gating aligned to the original antigen sequence indicated above as a scale. On top of scale, consensus epitopes summarize the minimal sequence needed for binding from each gated population.
(TIF)

Figure S4 **Epitope mapping of three antibodies towards FBXO28 using bacterial display.** FACS dot plots to the left show second sorting of a staphylococcal displayed FBXO28 peptide library. Colored bars to the right show sequences of collected clones from each gating aligned to the original antigen sequence indicated above as a scale. On top of scale, consensus epitopes summarize the minimal sequence needed for binding from each gated population.
(TIF)

Figure S5 **Epitope mapping of three antibodies towards IL17RA using bacterial display.** FACS dot plots to the left show second sorting of a staphylococcal displayed IL17RA peptide library. Colored bars to the right show sequences of collected clones from each gating aligned to the original antigen sequence indicated above as a scale. On top of scale, consensus epitopes summarize the minimal sequence needed for binding from each gated population.
(TIF)

Figure S6 **Epitope mapping of three antibodies towards TYMP using bacterial display.** FACS dot plots to the left show second sorting of a staphylococcal displayed TYMP peptide library. Colored bars to the right show sequences of collected clones from each gating aligned to the original antigen sequence indicated above as a scale. On top of scale, consensus epitopes summarize the minimal sequence needed for binding from each gated population.
(TIF)

Figure S7 **Epitope mapping of three antibodies towards PDXP using bacterial display.** FACS dot plots to the left show second sorting of a staphylococcal displayed PDXP peptide library. Colored bars to the right show sequences of collected clones from each gating aligned to the original antigen sequence indicated above as a scale. On top of scale, consensus epitopes summarize the minimal sequence needed for binding from each gated population.
(TIF)

Figure S8 **Epitope mapping of three antibodies towards C22orf29 using bacterial display.** FACS dot plots to the left show second sorting of a staphylococcal displayed C22orf29 peptide library. Colored bars to the right show sequences of collected clones from each gating aligned to the original antigen sequence indicated above as a scale. On top of scale, consensus epitopes summarize the minimal sequence needed for binding from each gated population.
(TIF)

Figure S9 **Epitope mapping of three antibodies towards FOXP2 using bacterial display.** FACS dot plots to the left show second sorting of a staphylococcal displayed FOXP2 peptide library. Colored bars to the right show sequences of collected clones from each gating aligned to the original antigen sequence indicated above as a scale. On top of scale, consensus epitopes summarize the minimal sequence needed for binding from each gated population.
(TIF)

Figure S10 **Epitope mapping of three antibodies towards ERBB2 using bacterial display.** FACS dot plots to the left show second sorting of a staphylococcal displayed ERBB2 peptide library. Colored bars to the right show sequences of collected clones from each gating aligned to the original antigen sequence indicated above as a scale. On top of scale, consensus epitopes summarize the minimal sequence needed for binding from each gated population.
(TIF)

Figure S11 **Western blot analysis of human tissue lysates using epitope-specific antibodies towards TYMP.** The same amount of epitope-specific antibody was used in each western blot analysis to compare the ability to detect TYMP in two human tissue lysates. Epitope-specific fractions of Antibody 1 (A), Antibody 2 (B) and Antibody 3 (C) towards TYMP. Marker (M), Liver (L) and Tonsil (T).
(TIF)

Table S1 **Epitope regions of antibodies on their antigens detected by bacterial surface display and peptide bead display.**
(XLSX)
