# Supplementary figures and images for: Parallel Immunizations of Rabbits Using the Same Antigen Yield Antibodies with Similar, but Not Identical, Epitopes
Source: PLoS One. 2012 Dec 19;7(12):e45817. doi: 10.1371/journal.pone.0045817 (PMC3526615; doi:10.1371/journal.pone.0045817)

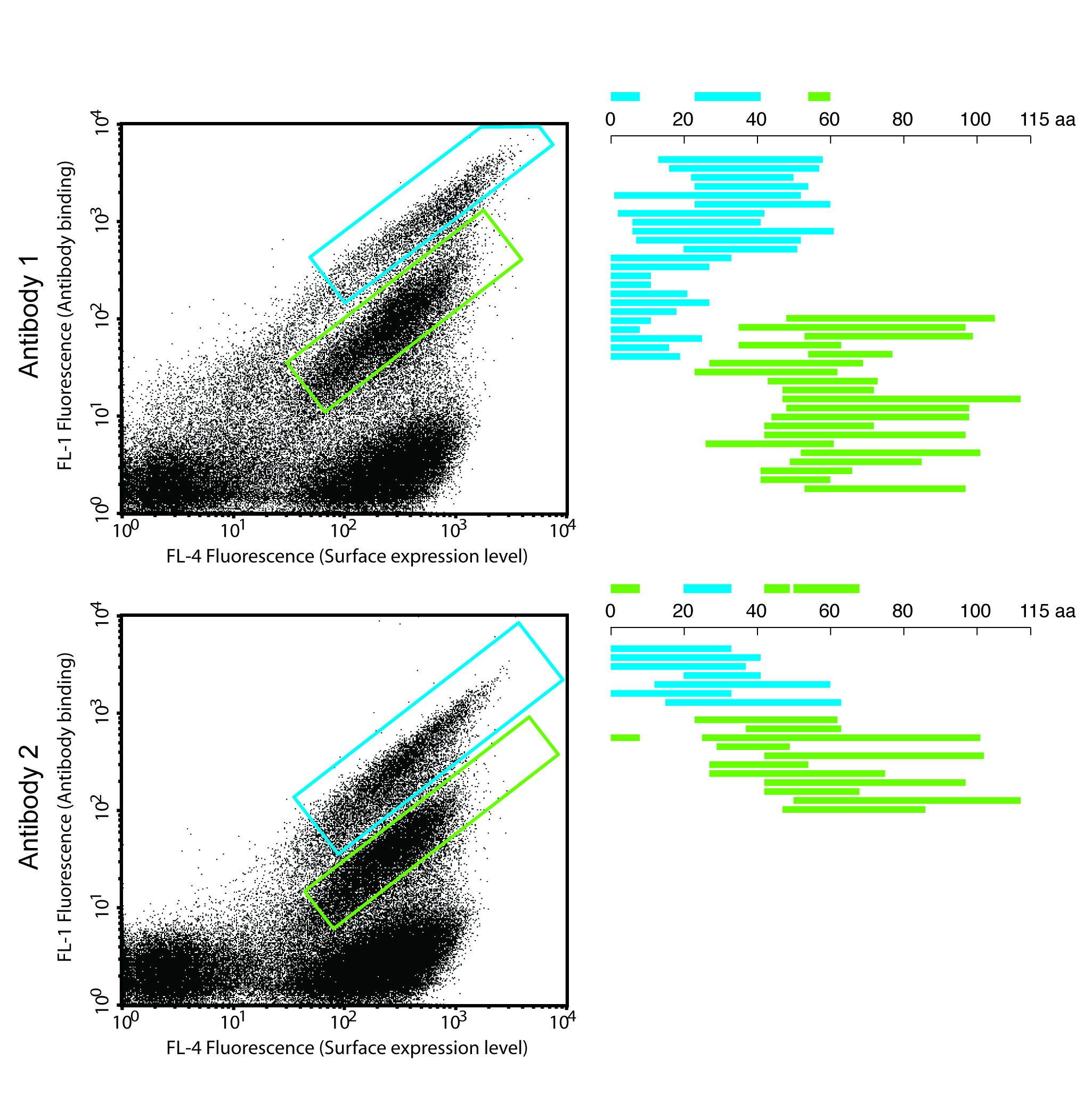

Supplement: Figure S1 — Epitope mapping of two antibodies towards HNRNPH2 using bacterial display. FACS dot plots to the left show second sorting of a staphylococcal displayed HNRNPH2 peptide library. Colored bars to the right show sequences of collected clones from each gating aligned to the original antigen sequence indicated above as a scale. On top of scale, consensus epitopes summarize the minimal sequence needed for binding from each gated population. (TIF) [file pone.0045817.s001.tif]

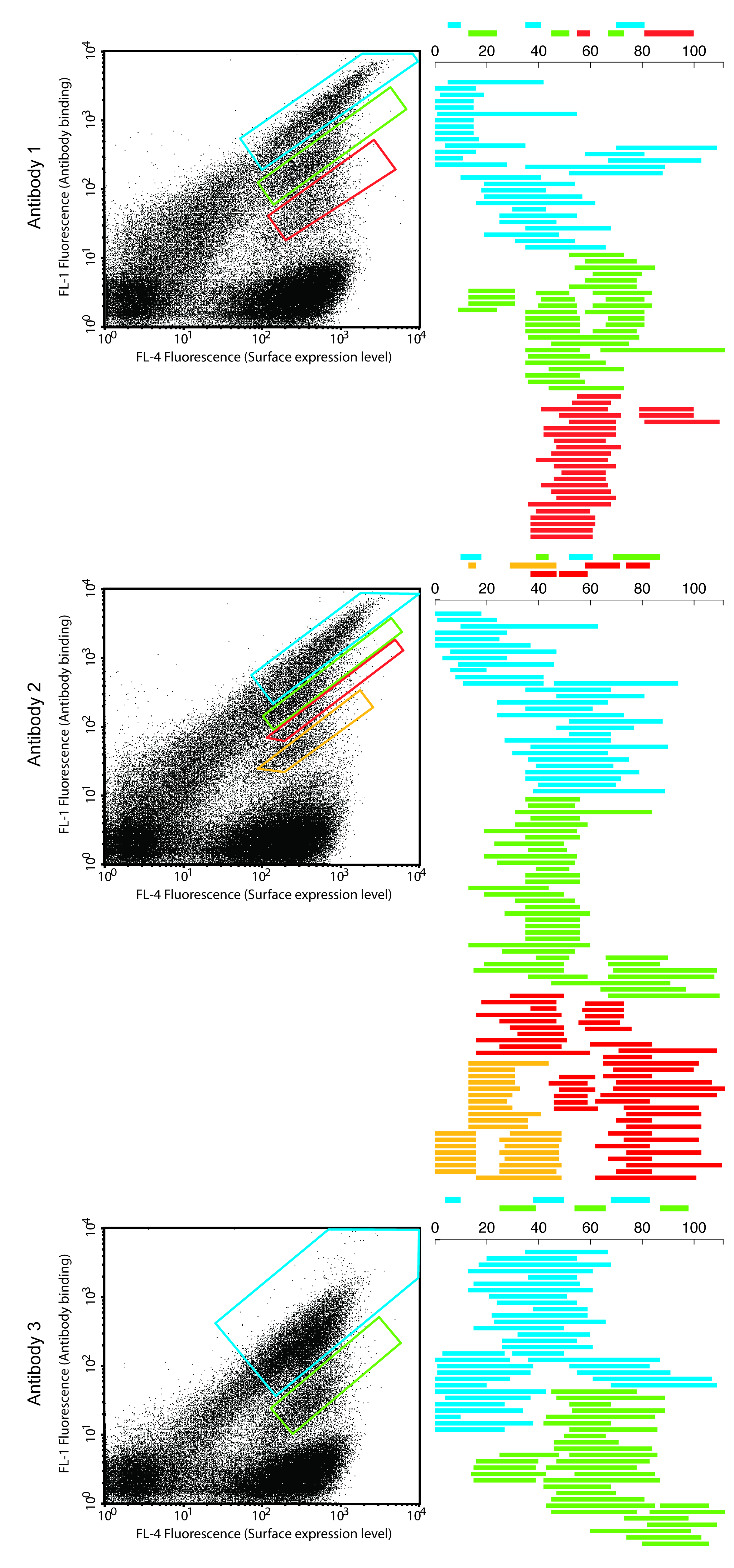

Supplement: Figure S2 — Epitope mapping of three antibodies towards SYNJ2BP using bacterial display. FACS dot plots to the left show second sorting of a staphylococcal displayed SYNJ2BP peptide library. Colored bars to the right show sequences of collected clones from each gating aligned to the original antigen sequence indicated above as a scale. On top of scale, consensus epitopes summarize the minimal sequence needed for binding from each gated population. (TIF) [file pone.0045817.s002.tif]

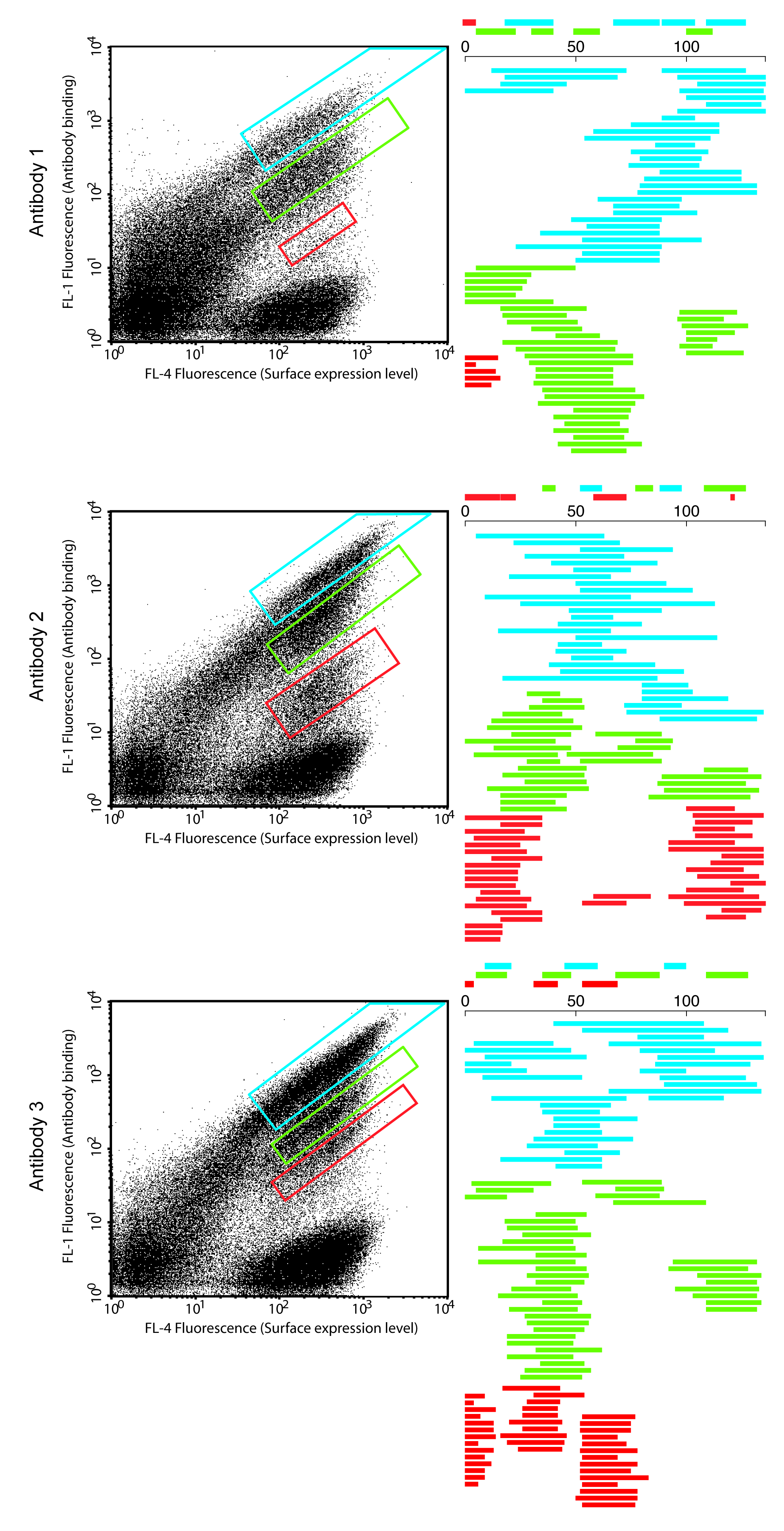

Supplement: Figure S3 — Epitope mapping of three antibodies towards RPS6KA5 using bacterial display. FACS dot plots to the left show second sorting of a staphylococcal displayed RPS6KA5 peptide library. Colored bars to the right show sequences of collected clones from each gating aligned to the original antigen sequence indicated above as a scale. On top of scale, consensus epitopes summarize the minimal sequence needed for binding from each gated population. (TIF) [file pone.0045817.s003.tif]

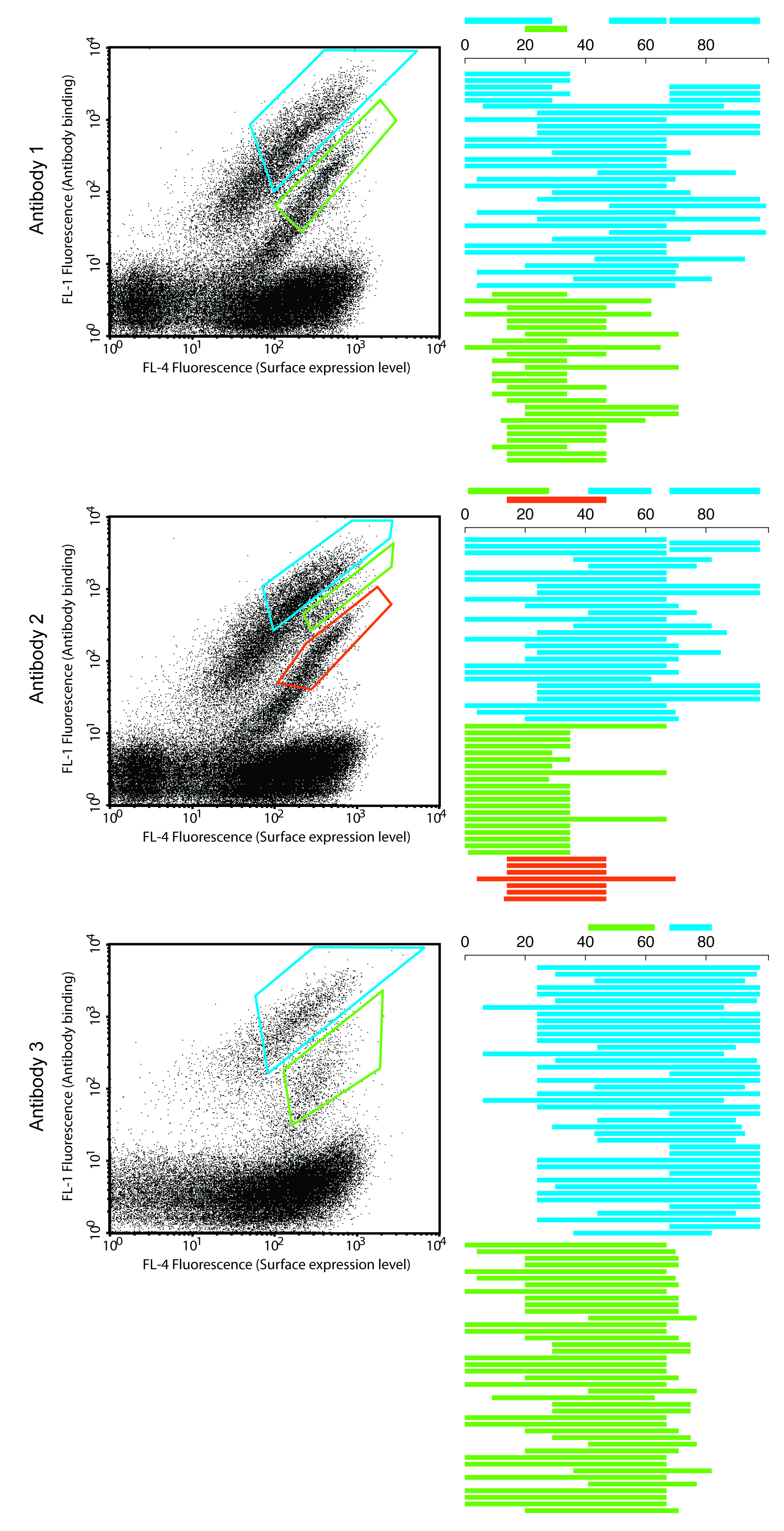

Supplement: Figure S4 — Epitope mapping of three antibodies towards FBXO28 using bacterial display. FACS dot plots to the left show second sorting of a staphylococcal displayed FBXO28 peptide library. Colored bars to the right show sequences of collected clones from each gating aligned to the original antigen sequence indicated above as a scale. On top of scale, consensus epitopes summarize the minimal sequence needed for binding from each gated population. (TIF) [file pone.0045817.s004.tif]

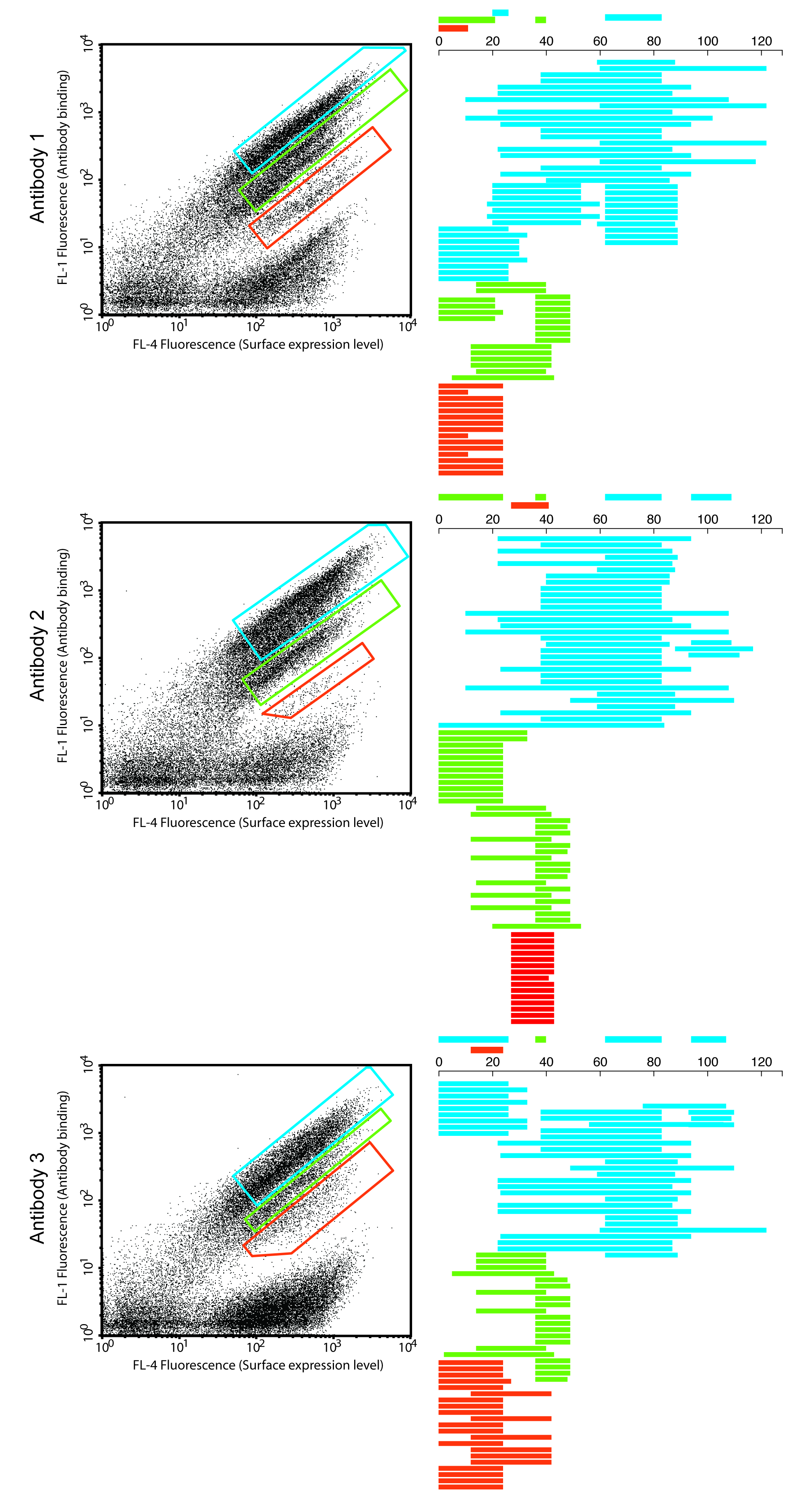

Supplement: Figure S5 — Epitope mapping of three antibodies towards IL17RA using bacterial display. FACS dot plots to the left show second sorting of a staphylococcal displayed IL17RA peptide library. Colored bars to the right show sequences of collected clones from each gating aligned to the original antigen sequence indicated above as a scale. On top of scale, consensus epitopes summarize the minimal sequence needed for binding from each gated population. (TIF) [file pone.0045817.s005.tif]

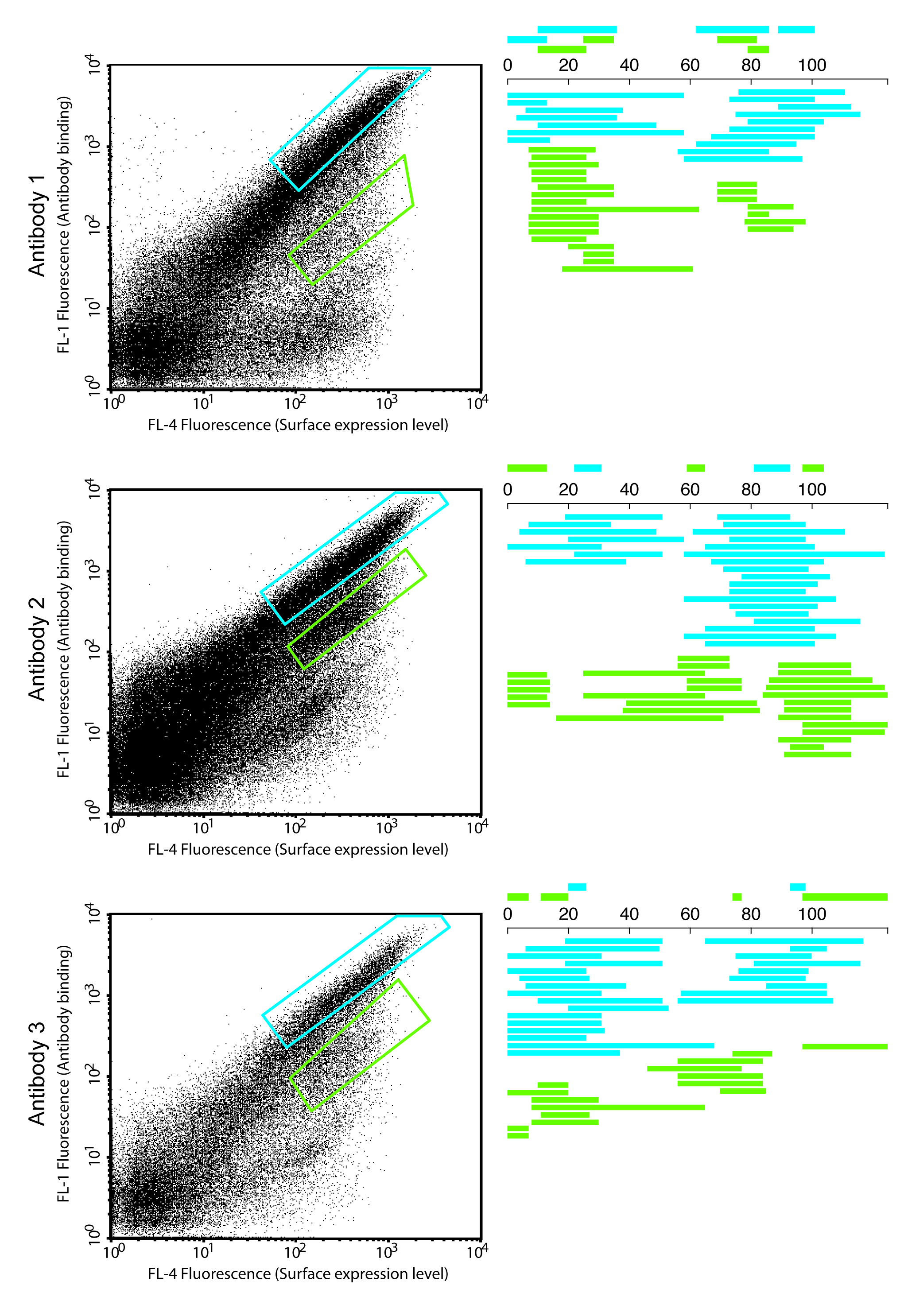

Supplement: Figure S6 — Epitope mapping of three antibodies towards TYMP using bacterial display. FACS dot plots to the left show second sorting of a staphylococcal displayed TYMP peptide library. Colored bars to the right show sequences of collected clones from each gating aligned to the original antigen sequence indicated above as a scale. On top of scale, consensus epitopes summarize the minimal sequence needed for binding from each gated population. (TIF) [file pone.0045817.s006.tif]

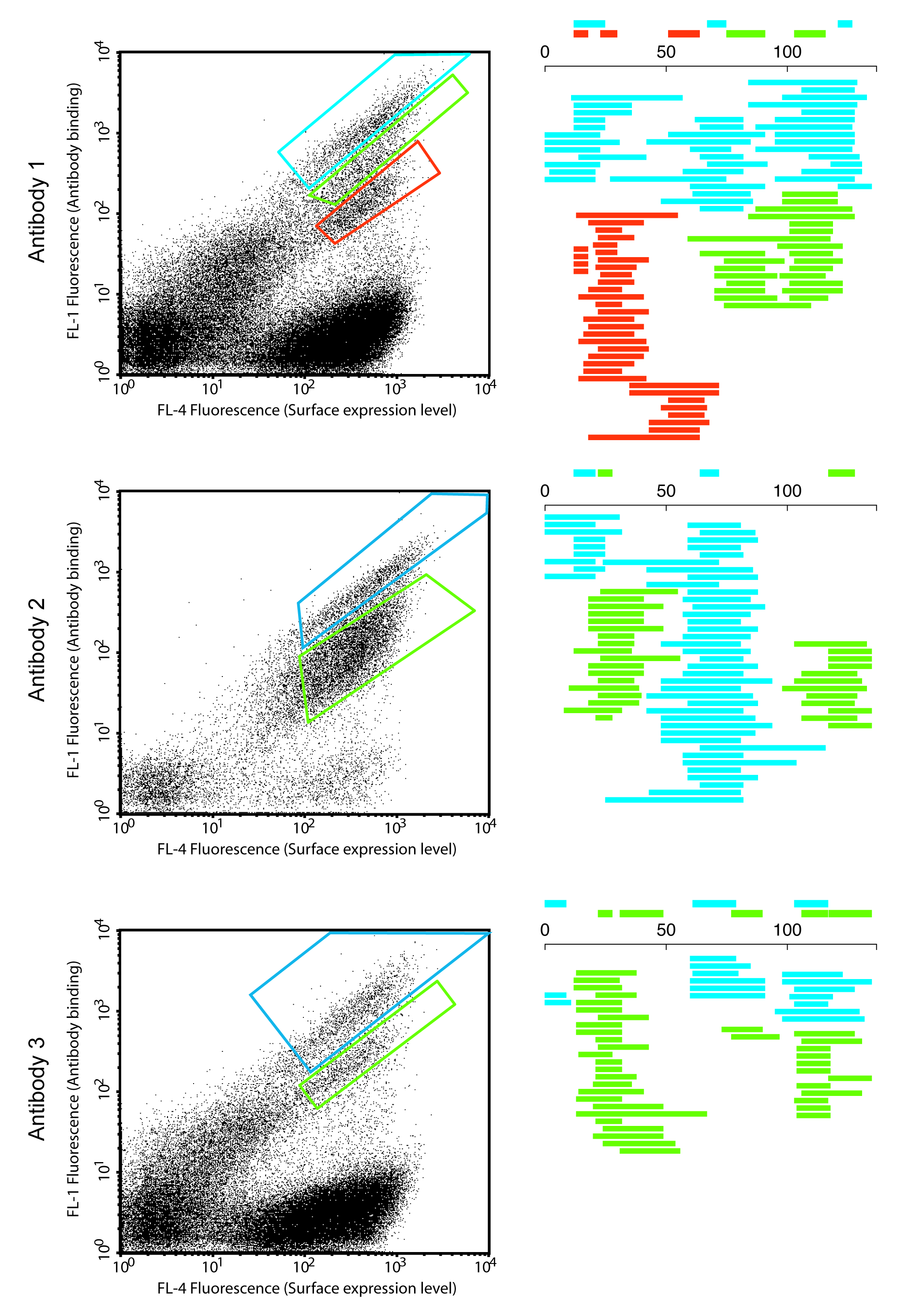

Supplement: Figure S7 — Epitope mapping of three antibodies towards PDXP using bacterial display. FACS dot plots to the left show second sorting of a staphylococcal displayed PDXP peptide library. Colored bars to the right show sequences of collected clones from each gating aligned to the original antigen sequence indicated above as a scale. On top of scale, consensus epitopes summarize the minimal sequence needed for binding from each gated population. (TIF) [file pone.0045817.s007.tif]

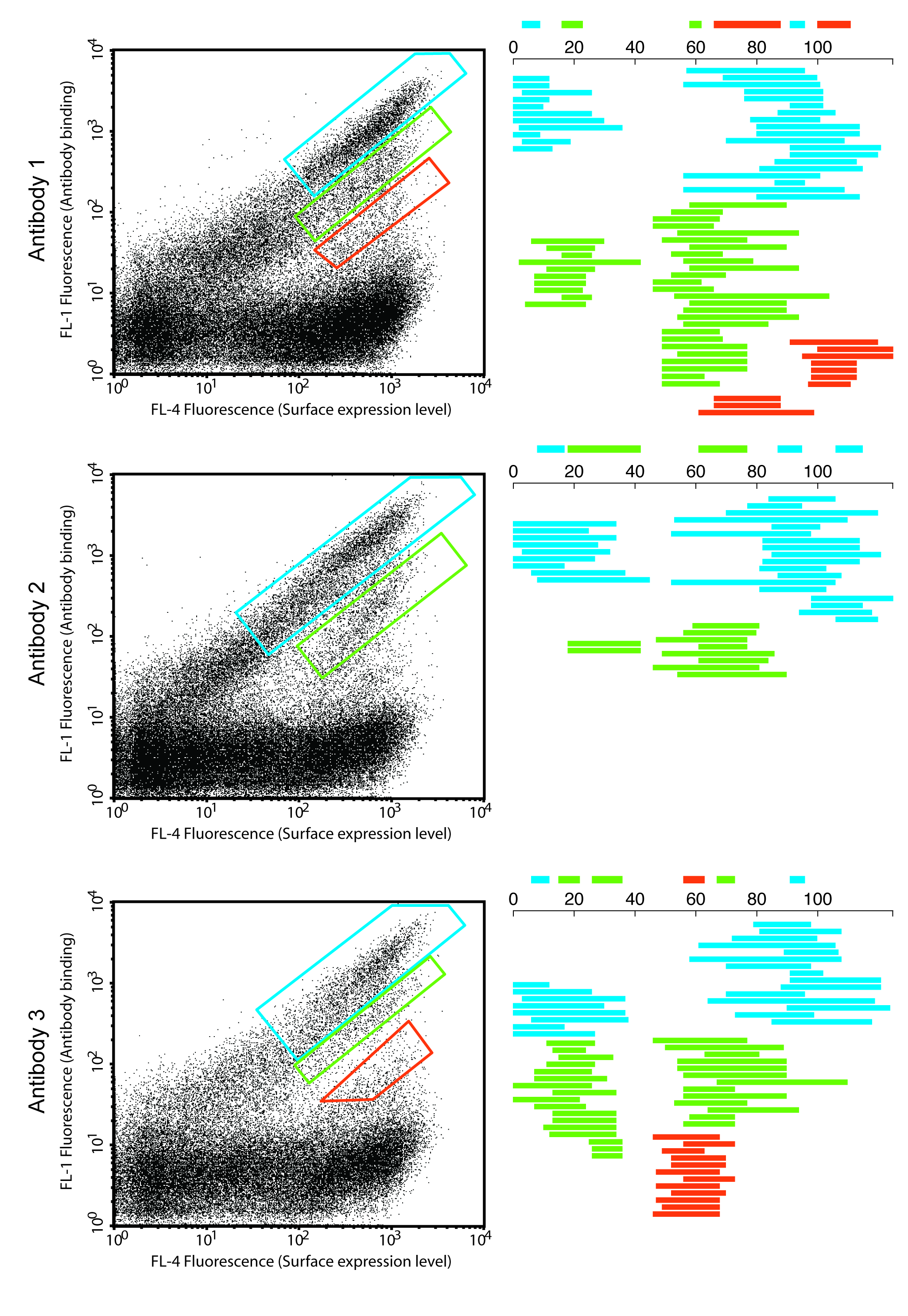

Supplement: Figure S8 — Epitope mapping of three antibodies towards C22orf29 using bacterial display. FACS dot plots to the left show second sorting of a staphylococcal displayed C22orf29 peptide library. Colored bars to the right show sequences of collected clones from each gating aligned to the original antigen sequence indicated above as a scale. On top of scale, consensus epitopes summarize the minimal sequence needed for binding from each gated population. (TIF) [file pone.0045817.s008.tif]

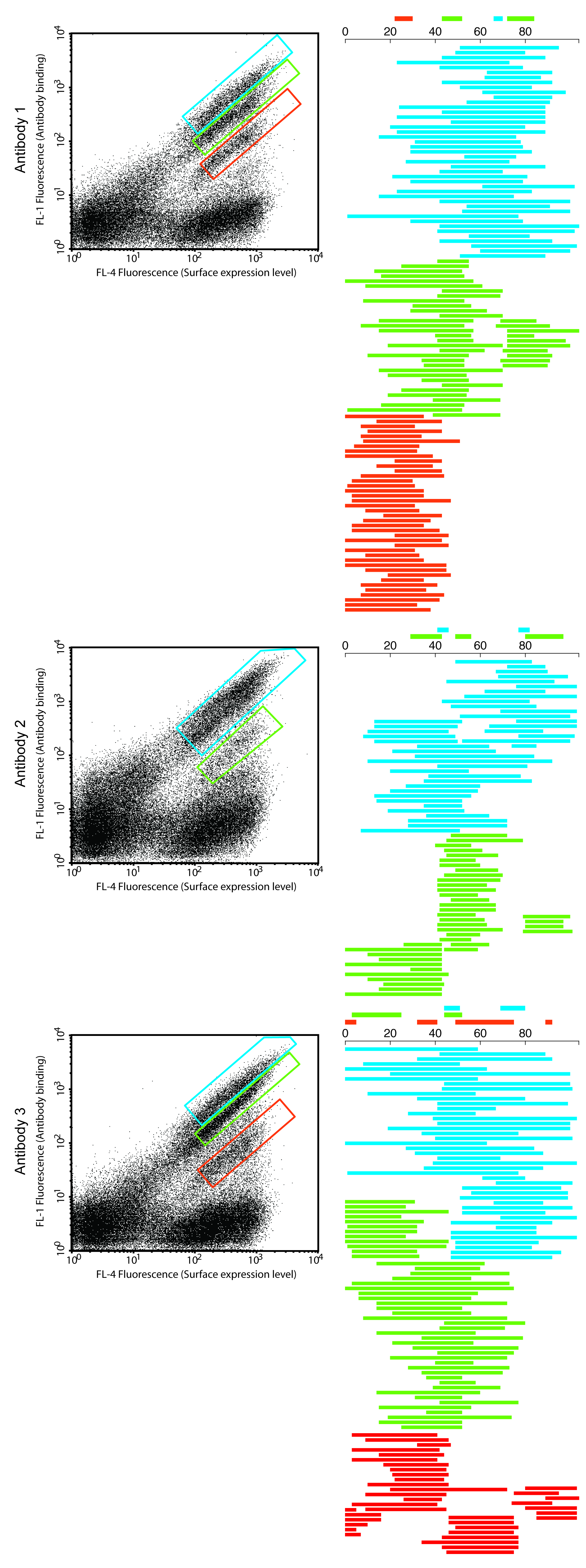

Supplement: Figure S9 — Epitope mapping of three antibodies towards FOXP2 using bacterial display. FACS dot plots to the left show second sorting of a staphylococcal displayed FOXP2 peptide library. Colored bars to the right show sequences of collected clones from each gating aligned to the original antigen sequence indicated above as a scale. On top of scale, consensus epitopes summarize the minimal sequence needed for binding from each gated population. (TIF) [file pone.0045817.s009.tif]

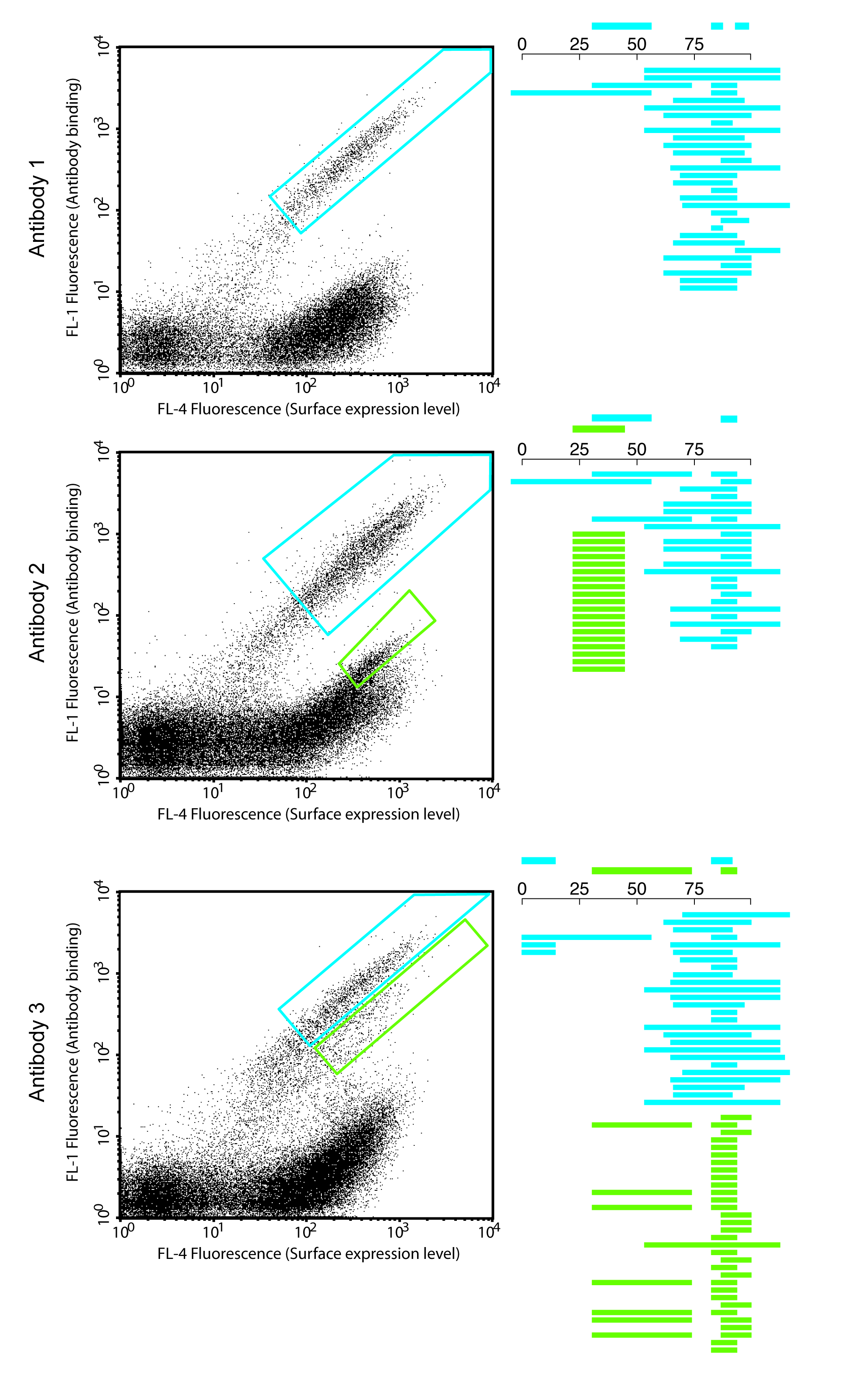

Supplement: Figure S10 — Epitope mapping of three antibodies towards ERBB2 using bacterial display. FACS dot plots to the left show second sorting of a staphylococcal displayed ERBB2 peptide library. Colored bars to the right show sequences of collected clones from each gating aligned to the original antigen sequence indicated above as a scale. On top of scale, consensus epitopes summarize the minimal sequence needed for binding from each gated population. (TIF) [file pone.0045817.s010.tif]

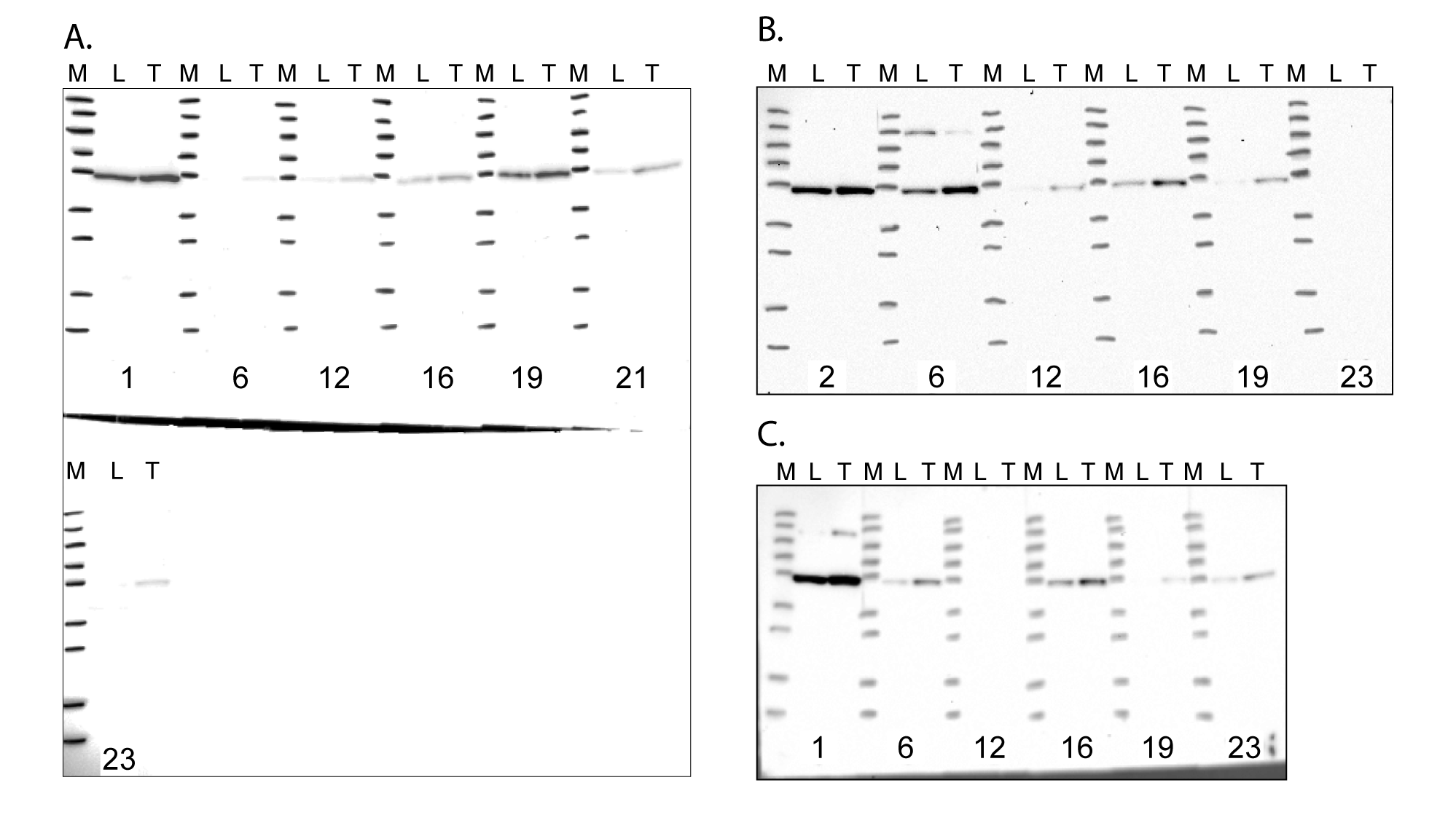

Supplement: Figure S11 — Western blot analysis of human tissue lysates using epitope-specific antibodies towards TYMP. The same amount of epitope-specific antibody was used in each western blot analysis to compare the ability to detect TYMP in two human tissue lysates. Epitope-specific fractions of Antibody 1 (A), Antibody 2 (B) and Antibody 3 (C) towards TYMP. Marker (M), Liver (L) and Tonsil (T). (TIF) [file pone.0045817.s011.tif]
